# Clinical and Prognostic Significance of Cell Sensitivity to Chemotherapy Detected In Vitro on Treatment Response and Survival of Leukemia Patients

**DOI:** 10.3390/jpm9020024

**Published:** 2019-05-07

**Authors:** Maria Kolesnikova, Aleksandra Sen’kova, Sofia Tairova, Viktor Ovchinnikov, Tatiana Pospelova, Marina Zenkova

**Affiliations:** 1 Department of therapy, hematology and transfusiology, Novosibirsk State Medical University, Krasny Prospect 52, 630091 Novosibirsk, Russia; marija.com.ka@mail.ru (M.K.); post_gem@mail.ru (T.P.); 2 Laboratory of nucleic acids biochemistry, Institute of Chemical Biology and Fundamental Medicine SB RAS, Lavrentieva ave. 8, 630090 Novosibirsk, Russia; alsenko@mail.ru; 3 Clinical and diagnostic laboratory, City Hematology Center, Polzunova Street 21, 630051 Novosibirsk, Russia; tairova.sofia@yandex.ru (S.T.); black_wizard@mail.ru (V.O.)

**Keywords:** leukemia, chemotherapy, drug sensitivity, multidrug resistance, P-glycoprotein

## Abstract

Multidrug resistance (MDR) is a major challenge in leukemia treatment. The objective of this study was to identity predictors of MDR to allow for rapid and economical assessment of the efficacy of planned antitumor therapy for leukemia patients. The study included 113 patients with acute and chronic leukemias. Prior to antitumor therapy, we measured the sensitivity of tumor cells of patients to the panel of chemotherapeutic drugs, together with *MDR1* mRNA and P-glycoprotein (P-gp) expression as one of the mechanisms of MDR, and compared these data with the response to therapy. The scales for leukemia patients according to therapy response, drug sensitivity of tumor cells, *MDR1* mRNA and P-gp levels, and the presence of unfavorable immunological and cytogenetic markers were introduced for subsequent correlation analysis. We show that the drug resistance of tumor cells of leukemia patients estimated in vitro at diagnosis correlates with a poor response to chemotherapy and is usually combined with aberrant and immature immunological markers, cytogenetic abnormalities, and a high expression of *MDR1* mRNA and P-gp. All together, these factors indicate unfavorable prognosis and low survival of leukemia patients. Thus, the sensitivity of tumor cells to chemotherapeutic drugs measured in vitro at diagnosis may have prognostic value for individual types of leukemia.

## 1. Introduction

Cancer is a leading cause of human deaths worldwide. The World Health Organization (WHO) has predicted that deaths from cancer will continue to rise, with an estimated 13.1 million deaths in 2030 [1,2]. Leukemia is the sixth most lethal cancer and accounts for 4% of all cancers. Recent surveys indicate that leukemia is the most common cause of cancer-related deaths in men under the age of 40, and approximately half of new cancer-related deaths among females have been attributed to leukemia [3].

Leukemia is a clonal malignant hematopoietic stem cell disorder and has four major subtypes: Acute myeloid leukemia (AML), acute lymphoblastic leukemia (ALL), chronic lymphocytic leukemia (CLL), and chronic myeloid leukemia (CML), which are very different in etiology, pathogenesis, clinical picture, and treatment approaches, and also have different prognoses and outcomes [4,5]. The main form of treatment for leukemia is chemotherapy. Drug resistance is often regarded as the main clinical obstacle to effective chemotherapy in patients diagnosed with leukemia [6]. Many resistance mechanisms have now been identified, and multidrug resistance (MDR) is considered the most important and prevalent reason for the failure of chemotherapy in the treatment of leukemia [7,8]. The main resistance mechanisms include overexpression of efflux pumps, such as P-glycoprotein (P-gp), multidrug resistance-associated proteins (MRP1, MRP2), lung resistance protein (LRP), and breast cancer resistance protein (BCRP); defective apoptotic machinery; increased drug metabolism due to altered molecular targets; enzyme-mediated drug resistance mechanisms, such as overexpression of glutathione S-transferase; microenvironmental resistance; and enhanced repair of drug-induced DNA damage [7]. One of the most studied is MDR mediated by efflux transporters of the ATP-binding cassette (ABC) superfamily, including the P-gp—membrane-associated drug efflux pump encoded by the *MDR1* gene in humans [9,10,11]. As a global problem, MDR limits the effective use of chemotherapeutic drugs, which leads to inadequate treatment and poor prognosis in leukemia patients.

Thus, MDR mechanisms are multiple and diversified and all of them are activated in response to anticancer therapy. However, all of the mechanisms cannot be identified in individual patients. The evaluation of a single mechanism of MDR, in particular MDR1/P-gp, may not reflect the true drug sensitivity or resistance in each patient. Therefore, it is more economical and faster to assess the total responsiveness to chemotherapeutic drugs, which are planned for use in the therapy of a particular patient. The identification of cell resistance to antitumor drugs and consequently an aggressive course of the disease at initial diagnosis allows one to modify standard therapy protocols or start with more aggressive complex cytotoxic regimens in second-line therapy [12,13].

However, despite the issue of drug resistance, in recent years, the prognosis of adult patients with acute leukemia has continuously improved due to the progress in leukemia treatment through the introduction of new diagnostic and therapeutic procedures [14,15,16]. Nevertheless, it is important to predict the patient’s response and efficacy of planned antitumor therapy at diagnosis and to identify unfavorable factors, such as MDR [17,18]. Despite the many approaches to overcome and reverse MDR [19,20,21], nowadays, reliable and low-cost detection techniques are needed to identify MDR at initial diagnosis, which can predict outcome and improve prognosis for leukemia patients [22,23,24].

Here, to analyze the relationships between MDR and the response to antitumor therapy, we evaluated the sensitivity of leukemic cells to chemotherapeutic drugs used in standard treatment schemes for 113 leukemia patients. In addition, *MDR1* mRNA and P-gp expression as well as immunological markers and cytogenetic abnormalities were analyzed at diagnosis for leukemia patients. The scales for leukemia patients according to the sensitivity of tumor cells to chemotherapeutics, therapy response, *MDR1* mRNA and P-gp levels, and the presence of unfavorable immunological and genetic markers were designed for subsequent correlation analysis. We show that the drug resistance of tumor cells of leukemia patients estimated in vitro at diagnosis correlates with a poor response to chemotherapy, usually combined with aberrant and immature immunological markers, cytogenetic abnormalities, and elevated *MDR1* mRNA and P-gp expression. All together, these factors indicate unfavorable prognosis and low survival of patients, especially with acute leukemia. Thus, the analysis of drug sensitivity or resistance of tumor cells can be used in routine clinical practice as a prediction of treatment response and outcome in leukemia patients.

## 2. Materials and Methods

### 2.1. Patients

The study included 113 hematological patients: Forty-nine patients with AML, 16 patients with ALL, 43 patients with CLL, and 5 patients with CML. In all leukemia patients, the diagnosis was confirmed by morphological assessment of peripheral blood smears and bone marrow aspirates/biopsy, flow cytometry, cytogenetic analysis, and standard laboratory and instrumental studies included in the diagnostic protocol of patients with leukemia. Demographic and clinical data—including age, gender, date of presentation, and clinical and laboratory parameters of leukemia at the time of diagnosis and during follow-up—were collected. Additional patient information, including time of relapse and mortality, were obtained from medical records or from institutional databases containing information regarding disease status, complications, and survival.

#### 2.1.1. Inclusion Criteria

Patients with acute and chronic leukemia based on clinical, morphological, and genetic markers were included in the study to demonstrate differences between the applicability of the studied approach in relation to leukemia with different origins and clinical courses. All patients with acute leukemia were randomized into two groups: “Primary” and “secondary”. Patients with acute leukemia newly diagnosed on admission were considered to be “primary” patients. Patients with acute leukemia who had previously received antitumor chemotherapy, patients with acute leukemia as a secondary malignancy, and as a result of transformation of other hematological disorders were considered to be “secondary” patients.

#### 2.1.2. Exclusion Criteria

Patients presenting with hematological disorders (e.g., cytopenia, anemia) as a result of other non-malignant causes, patients with HIV, hepatitis B and C, and tuberculosis were excluded from the study.

### 2.2. Collection of Peripheral Blood and Bone Marrow Samples

Peripheral blood (PB) from leukemia patients was obtained in BD Vacutainer by venipuncture (BD Biosciences, Franklin Lakes, NJ, USA). Bone marrow (BM) samples were collected in ethylenediaminetetraacetic acid (EDTA)- or heparinised-glass tubes by sternal puncture or iliac crest aspiration/biopsy using a jamshidi needle. The PB and BM samples were transported to the laboratory for analysis within 1 to 3 h.

Human samples of PB and BM were obtained at the time of diagnosis before chemotherapy with the signed informed consent of the leukemia patients and with ethical approval from the Institutional Review Board of Novosibirsk State Medical University (No. 80/2015) prior to commencement of the study in accordance with the ethical principles of the Declaration of Helsinki in the revised version (2013) and in compliance with national laws [25].

### 2.3. Cell Isolation and Culture

The PB and BM samples were processed within a few hours following collection. Leukemia cells were isolated from the PB and BM of patients by centrifugation in lymphocyte separation medium (MP Biomedicals, Santa Ana, CA, USA), according to the manufacturer’s instructions. Following isolation, cells were cultured in the Iscove’s Modified Dulbecco’s Medium (IMDM) supplemented with 10% fetal bovine serum, 1% antibiotic antimycotic solution (10,000 μg/mL streptomycin, 10,000 IU/mL penicillin, and 25 μg/mL amphotericin; ICN, Eschwege, Germany) at 37 °C in a 5% CO_2_ humidified atmosphere. Sufficient amounts and good maintenance of isolated leukemia cells in vitro was a necessary condition for subsequent analysis.

### 2.4. Drug Sensitivity Estimation (Water Soluble Tetrazolium (WST)-Test)

For drug sensitivity estimation, leukemia cells isolated from PB and/or BM of leukemia patients were used. All cell lines were cultured in IMDM as described above. Cells were plated in 96-well flat-bottom plates at a density of 0.5 × 10^5^ to 2 × 10^5^ cells per well. Cells were incubated either in medium alone (control) or exposed to increasing concentrations of chemotherapeutic drugs for 72 h at 37 °C. Doxorubicin (0–2 μM), daunorubicin (0–2 μM), cytarabine (0–82 μM), and vincristine (0–5 μM) (all obtained from TEVA, Rehovot, Israel) were stored according to the manufacturer’s recommendations and diluted immediately prior to use.

Following incubation with chemotherapeutic drugs, 10 μL solution of 0.5 mg/mL water soluble tetrazolium WST-1 (Roche, Basel, Switzerland) was added to each well, and cells were incubated for 3 h at 37 °C in a CO_2_ incubator [26,27]. Following this, the absorbance was measured spectrophotometrically using Multiscan RC (Labsystems, Vantaa, Finland) at 450 and 620 nm. Then, the concentration of the chemotherapeutic drug that caused the death of 50% of tumor cells (IC_50_) was calculated. The IC_50_ values were obtained by fitting dose-response curves according to the following exponential decay equation using non-linear regression: y = y_0_ + A_1_e^−(x − x0)/t1^, where x is the concentration of the substance and y = 50%. The IC_50_ values were calculated from three independent measurements. The significance of differences was analyzed using an unpaired Student’s *t*-test.

For each patient, IC_50_ values for a number of cytotoxic drugs were defined. For AML and CML patients, sensitivity to doxorubicin, daunorubicin, and cytarabine was estimated; for ALL patients, sensitivity to doxorubicin, daunorubicin, cytarabine, and vincristine was estimated; and for CLL patients, sensitivity to doxorubicin and vincristine was estimated. A list of chemotherapeutics for in vitro study was determined in accordance with the list of drugs used for antitumor therapy of particular hematological diseases in clinical practice. It is known that the treatment standard for patients with CLL is the regimen, which includes rituximab, fludarabine, and cyclophosphamide (R-FC). It should be noted that in our study, most CLL patients were presented with CLL/small lymphocytic lymphoma (CLL/SLL), including both P53 mutations and severe comorbidity. For this reason, they received antracycline-based treatment protocols in accordance with official recommendations and literature data [28,29,30], which contain doxorubicin and vincristine (COP—cyclophosphamide, vincristine (earlier oncovin), prednisolone; CHOP—cyclophosphamide, doxorubicin (earlier hydroxydaunorubicin), vinctistine (earlier oncovin), prednisolone; R-COP—rituximab, cyclophosphamide, vinctistine (earlier oncovin), prednisolone; R-CHOP—rituximab, cyclophosphamide, doxorubicin (earlier hydroxydaunorubicin), vinctistine (earlier oncovin), prednisolone; miniCHOP and R-miniCHOP—the same drugs in reduced doses; R-CHOEP—rituximab, cyclophosphamide, doxorubicin (earlier hydroxydaunorubicin), vinctistine (earlier oncovin), prednisolone, etoposide) (Table S3). Therefore, in this study, we evaluated the sensitivity of CLL cells to these drugs.

### 2.5. Immunophenotyping

Immunophenotyping of leukemia patients was performed on PB and/or BM samples at diagnosis. Reactivity with monoclonal antibodies directed against a number of antigens was evaluated by flow cytometry.

The PB and/or BM samples were incubated with VersaLyse Lysing Solution (A09777, Beckman Coulter, Brea, CA, USA) at 37 °C for 2 min. Leukemic cells were then isolated by centrifugation at 400 × g for 3 min and 30 s. The supernatant was removed, and the cells were washed and fixed with IOTest 3 Fixative Solution (A07800, Beckman Coulter), and incubated with monoclonal antibodies. The resulting samples were assayed using a flow cytometer Cytomics FC500 (Beckman Coulter) and CXP Software 3.16. At least 3 × 10^6^ cells were analyzed from each sample.

Expression of CD2, CD3, cCD3, CD4, CD5, CD7, CD10, CD11c, CD13, CD14, CD15, CD16, CD19, CD20, sCD22, CD23, CD25, CD26, CD30, CD33, CD34, CD38, CD43, CD45, CD56, CD61, CD64, CD65, CD71, cCD79a, CD103, CD117, CD138, HLA-DR, TCR, FMC7, TdT, cMPO, Ki-67, bcl-2, and ZAP-70 in leukemia cells was analyzed. The full list of monoclonal antibodies used for immunophenotyping of leukemia patients can be found in Table S1.

The surface markers were considered positive when ≥20% of the blasts expressed the antigen. The cytoplasmic positive expression was based on ≥10% reactive blasts. The immunologic definition in leukemia lineage was based on the WHO classification of tumors of hematopoietic and lymphoid tissues [4,5].

### 2.6. Cytogenetic Analyses

Cytogenetic abnormalities in the leukemic cells of patients were studied by karyotyping and fluorescence in situ hybridization (FISH) analysis.

Cytogenetic analysis was performed on direct and 24 h unstimulated PB and/or BM cell cultures using standard procedures. The GTG-banding chromosomes were classified according to the International System for Human Cytogenetic Nomenclature 2013 [31], and karyograms were generated using the LUCIA Cytogenetics 2.0. Successful cytogenetic analysis required the detection of at least two cells with the same structural changes or chromosomal gain, three or more cells with the same chromosomal loss, or at least 20 metaphases without clonal changes.

The following gene abnormalities were analyzed in leukemia patients: Translocations t(15;17)(q24;q21), t(9;22)(q34;q11), t(12;21)(p13;q22), t(8;14)(q24;q32), t(8;21)(q21;q22), and t(16;16)(p13;q22); locus monosomy of P53(17p13.1); isochromosome 17 i(17)(q11); MLL(11q23.3); EGR1(5q31.2) (deletion 5); RELN(q22); TES(q31); EVI1(3q26.2); KMT2A(11q23); AFF1(4q21.22); IGH(14q32); BCL6(3q27); E2A(19p13.3); AML1(21q22.12); cMYC(8q24); MYB(6q23.3); TRA/D(14q11); FGFR1(8p11); TAS2R1(5p15.31); ATM(11q22.3); and trisomies 6, 7, 8, 9, 12, 17, and 21.

Metaphase FISH analysis was performed on cytogenetic preparations obtained from BM cells of leukemia patients. Direct labelling locus specific FISH probes (Abbott Vysis, Des Plaines, IL, USA) were used for bcr/abl gene fusion and MLL and C-MYC gene rearrangements according to the manufacturer’s recommendations. The size of genetically abnormal clones was determined following the analysis of at least 100 successfully hybridized cells.

### 2.7. Multidrug Resistance Evaluation

Multidrug resistance evaluation was performed on PB and/or BM samples of leukemia patients (AML, ALL, and CLL) using quantitative real-time reverse transcription-polymerase chain reaction (real-time qPCR) and immunocytochemistry. Real-time qPCR was used to evaluate the expression of *MDR1* mRNA in leukemic cells. Immunocytochemistry was used to evaluate the amount of P-gp in leukemic cells. Patients with CML were excluded from this part of the study because this disorder was mainly presented by chronic myelofibrosis, which did not allow isolation of a sufficient amount of tumor cells.

#### 2.7.1. Real-time qPCR

Total RNA from leukemic cells was extracted using TRIzol Reagent (Invitrogen, Carlsbad, CA, USA) according to the manufacturer’s instructions. The RNA quantification was performed with SYBR Green-based real-time qPCR using IQ5 Cycler (Bio-Rad, Hercules, CA, USA). The cDNA was amplified in a total volume of 20 µL containing 2 µL of cDNA template, RNA specific primers (1 µM), and BioMaster HS-qPCR SYBR Blue master mix with SYBR Green I fluorescent dye (Biolabmix, Novosibirsk, Russia). The following primers were used: MDR1_F: 5’-AGAGAATCCCCTCCAGATAAGA–3’ and MDR1_R: 5’-AAGCCTATTCCATTTTGAACTTTCT-3’, GAPDH_F: 5’-GTGAAGGTCGGAGTCAAC-3’ and GAPDH_R: 5’-TGGAATTTGCCATGGGTG-3’. All measurements were done in triplicate. The amount of RNA was calculated from the number of cycles using standard curves and the results were normalized to glyceraldehyde 3 phosphate dehydrogenase (GAPDH). The obtained PCR data were analyzed using Bio-Rad iQ5 v.2.0 software.

#### 2.7.2. Immunocytochemistry

For immunocytochemistry, historical material of PB and/or BM smears of leukemia patients was used. Samples were incubated with anti-P-gp antibody (EPR10364-57) (ab170904; Abcam, Cambridge, UK) according to the manufacturer’s instructions. For detection and visualization, a UltraVision Quanto Detection System (Thermo Fisher Scientific, Waltham, MA, USA) and Permanent Fast Red Quanto Substrate System (Thermo Fisher Scientific) were used. Finally, slides were counterstained by Giemsa–Romanovsky staining and visualized under the microscope Axiostar Plus equipped with an Axiocam MRc5 digital camera (Carl Zeiss Microscopy GmbH, Jena, Germany).

Red membrane staining was considered positive for P-gp. Carcinoma KB-8-5 cells with confirmed and stable P-gp expression served as a positive control. Chronic lymphocytic leukemia cells with an absence of *MDR1* mRNA and P-gp expression confirmed by real-time qPCR and immunocytochemistry, respectively, were used as a negative control. The index of P-gp staining (In(P-gp)) used for assessment of PB and/or BM smears was calculated as follows: In(P-gp) = (P-gp-positive cell number/total cell number) × 100%, where the total cell number was at least 100 cells for each sample.

### 2.8. Statistical Analysis

Correlation analysis was performed using Spearman’s rank order correlation coefficient, r_s_ (hereinafter *r*), which is a statistical measure of the strength of the relationship between paired data. The relationship between studied parameters (variables) was estimated, taking the values of the Spearman’s coefficient equal to 0.01 to 0.29 as indicative of a weak positive correlation, 0.30 to 0.69 as a moderate positive correlation, and 0.70 to 1.0 as a strong positive correlation. The following variables were analyzed: Sensitivity of leukemia cells to cytotoxic drugs, response of leukemia patients to chemotherapy, immunological markers, cytogenetic abnormalities, and *MDR1* mRNA and P-gp expression in leukemia cells.

Overall survival was estimated using the Kaplan–Meier method, and comparisons between groups were done using the log-rank test.

All other results are presented as the means ± standard error of the mean. An unpaired Student’s t-test was performed to analyze drug sensitivity data, *MDR1* mRNA, and P-gp quantification data. For all tests, values of *p* ≤ 0.05 were considered statistically significant. All statistical analyses were performed with MS Excel, OriginPro 7.5, and Statistica 10.0.

## 3. Results

### 3.1. Range of Patients with Acute and Chronic Leukemia according to the Sensitivity of Tumor Cells to Chemotherapeutic Drugs

The study included 113 hematological patients: Forty-nine patients with AML (30 primary, 19 secondary), 16 patients with ALL (11 primary, 5 secondary), 43 patients with CLL, and 5 patients with CML. The average age and gender distribution of patients are presented in Table S2.

Sensitivity of tumor cells (IC_50_ values) to a number of chemotherapeutic drugs was evaluated using the WST-test for all leukemia patients at diagnosis before the treatment start. The WST-assay method was applied in the study because, based on the estimation of mitochondrial dehydrogenase activity in cells, it is also useful as an indicator of cell viability and toxicity [32,33,34], and nowadays WST assay is widely used for the screening of drug responsiveness of cancer cells with different origins [35,36,37].

Selection of the drug panel for the study was based on a set of drugs used in the first-line therapy of individual types of leukemia (see Section 2). The obtained IC_50_ values for the studied chemotherapeutics for each patient as well as treatment regimens and therapy response are listed in Table S3. It can be clearly seen that the initial sensitivity of tumor cells to chemotherapeutic drugs vary significantly even for the primary diagnosed leukemia patients.

Then, we scaled the drug sensitivity of leukemia cells for subsequent correlation analysis based on IC_50_ values: Scale 1 corresponds to a high sensitivity of tumor cells, and scales 2 and 3 correspond to a moderate and low (resistance) sensitivity of leukemic cells, respectively. It should be noted that scales for different types of leukemia vary significantly (Table 1). As an example, scale 3 (resistance) was assigned to patients whose cells were characterized by IC_50_ values for doxorubicin ˃ 1 μM for AML, ˃0.1 μM for ALL, and ˃0.5 μM for CLL and CML (Table 1). In this way, all leukemia patients were divided into groups according to the sensitivity of their tumor cells to cytotoxic drugs based on IC_50_ values.

### 3.2. Range of Patients with Acute and Chronic Leukemia according to the Therapy Response, Immunological Markers, and Cytogenetic Abnormalities in Tumor Cells

All leukemia patients, along with standard clinical and laboratory tests, were initially analyzed using an established panel of immunological and cytogenetic markers to make a correct diagnosis and to carry out individual risk stratification for the selection of the most appropriate therapeutic procedures.

The most important parameter for the assessment of the efficacy of prescribed antitumor therapy is the therapy response of leukemia patients. In acute leukemia, the therapy response was scaled based on the number of blasts in the BM after 1 to 2 courses of chemotherapy, which corresponds to standard international criteria [38]: 1, remission (˂5% blasts); 2, relapse with subsequent remission (5–20% blasts); and 3, relapse with subsequent resistance or initial resistance (˃20% blasts). The therapy response for patients with chronic leukemia was scaled based on complex clinical and laboratory data: 1, remission or partial remission; 2, stabilization; and 3, progression or initial resistance. In this way, all leukemia patients were divided into three groups based on their response to chemotherapy (Table S3).

According to the results of the performed immunophenotyping and cytogenetic analysis (for details, see Section 2), all patients were divided into groups based on the positive or negative expression of immunological markers and on the presence or absence of cytogenetic abnormalities in leukemic cells: 1, negative expression of immunological markers and absence of cytogenetic abnormalities in leukemic cells; and 2, positive expression of immunological markers and presence of cytogenetic abnormalities in leukemic cells (Table S4).

### 3.3. Range of Patients with Acute and Chronic Leukemia according to the Expression of MDR1 mRNA and P-Glycoprotein in Tumor Cells

Low sensitivity or resistance of leukemic cells to chemotherapeutics can be the result of overexpression of the *MDR1* gene followed by incorporation of P-gp, which is encoded by the *MDR1* gene, into the membrane of tumor cells. The expression levels of *MDR1* mRNA and P-gp were measured by real-time qPCR and immunocytochemistry, respectively (for details, see Section 2).

**Figure 1:**
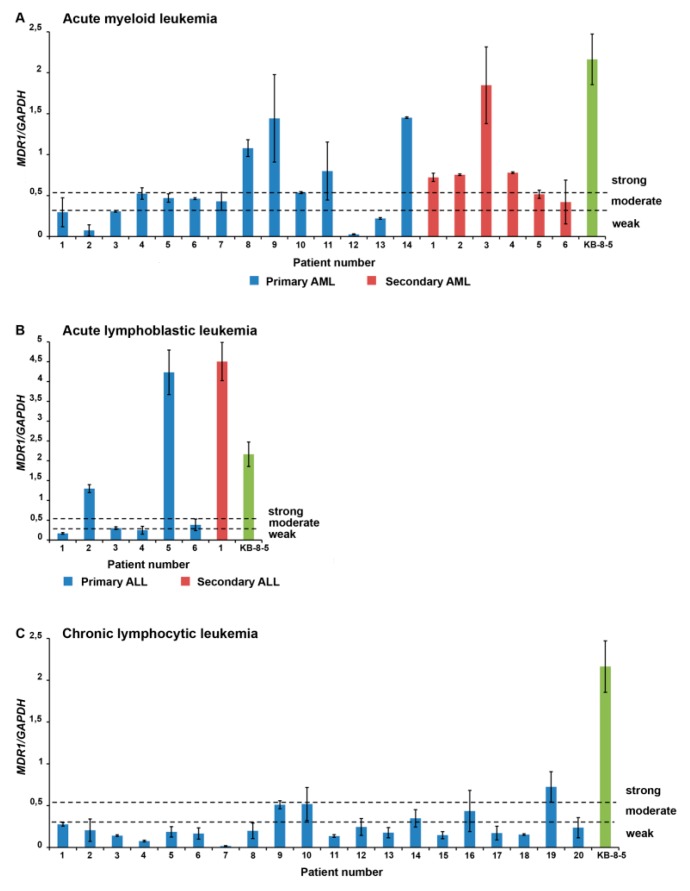
The *MDR1* mRNA expression levels in tumor cells of leukemia patients. The following scale was used for the distribution of patients with acute myeloid leukemia (**A**), acute lymphoblastic leukemia (**B**), and chronic lymphocytic leukemia (**C**) in accordance with *MDR1* mRNA expression levels in tumor cells: weak expression corresponds to 0–15% of the level of KB-8-5 cells, moderate expression corresponds to 15–25% of the level of KB-8-5 cells, and strong expression corresponds to >25% of the level of KB-8-5 cells.

The *MDR1* mRNA expression levels for each patient are shown in Figure 1. High levels of *MDR1* mRNA predominate among patients with secondary acute leukemia (secondary AML and secondary ALL), whereas tumor cells of patients with primary acute leukemia (primary AML and primary ALL) and chronic leukemia (CLL) are characterized by moderate and low levels of *MDR1* mRNA (Figure 1).

**Figure 2:**
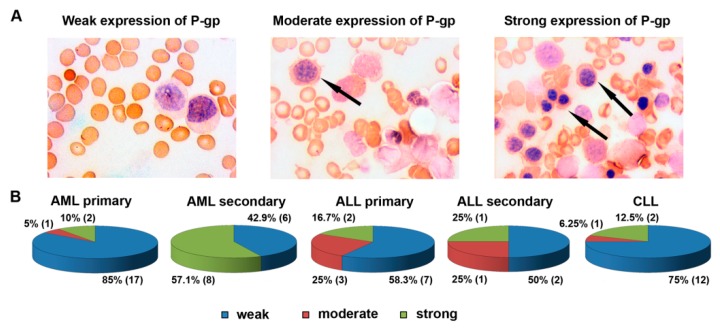
P-glycoprotein expression in tumor cells of leukemia patients. Typical images of immunocytochemical staining of bone marrow smears with anti-P-glycoprotein monoclonal antibodies (**A**) and patient distribution according to P-glycoprotein expression levels (**B**) is presented for patients with acute and chronic leukemia. Representative images of bone marrow smears are presented for patients with primary (left panel) and secondary (central and right panel) acute myeloid leukemia. Black arrows indicate P-glycoprotein positive tumor cells. The number of patients in each group is indicated in parentheses.

Patient distribution according to P-gp levels in tumor cells is presented in Figure 2. Similar to *MDR1* mRNA, much stronger expression of P-gp predominates among patients with secondary acute leukemia compared to primary acute leukemia and chronic leukemia (Figure 2). Thus, high expression levels of *MDR1* mRNA and P-gp, reflecting one of the mechanisms of MDR, were observed in leukemic patients with complicated hematological anamnesis.

Then, based on the *MDR1* mRNA and P-gp expression levels in leukemic cells, the following scales for subsequent correlation analysis were used: 1, weak expression of *MDR1* mRNA (0–15% of the level of KB-8-5 cells) and P-gp (0–5% P-gp-positive cells); 2, moderate expression of *MDR1* mRNA (15–25% of the level of KB-8-5 cells) and P-gp (5–10% P-gp-positive cells); and 3, strong expression of *MDR1* mRNA (>25% compared with KB-8-5 expression level) and P-gp (>10% P-gp-positive cells).

### 3.4. Correlations of Drug Sensitivity of Tumor Cells with Therapy Response, Immunological Markers, and Cytogenetic Abnormalities in Patients with Acute and Chronic Leukemia

The correlations of all measured and recorded parameters were analyzed using nonparametric correlation analysis with the calculation of Spearmen’s correlation coefficients (*r*). In correlation analysis, acute and chronic leukemias as well as myeloid and lymphoid leukemias were analyzed separately, and acute leukemias were divided into primary and secondary. It should be stressed that in all cases studied, we observed strong or moderate correlations between high or moderate drug sensitivity of tumor cells (score 1 or 2), good therapy response (score 1 or 2), absence of genetic abnormalities (score 1), and unfavorable immunological markers (score 1); and drug resistance of tumor cells (score 3), poor therapy response (score 3), presence of genetic abnormalities (score 2), and unfavorable immunological markers (score 2).

#### 3.4.1. Primary AML

**Figure 3:**
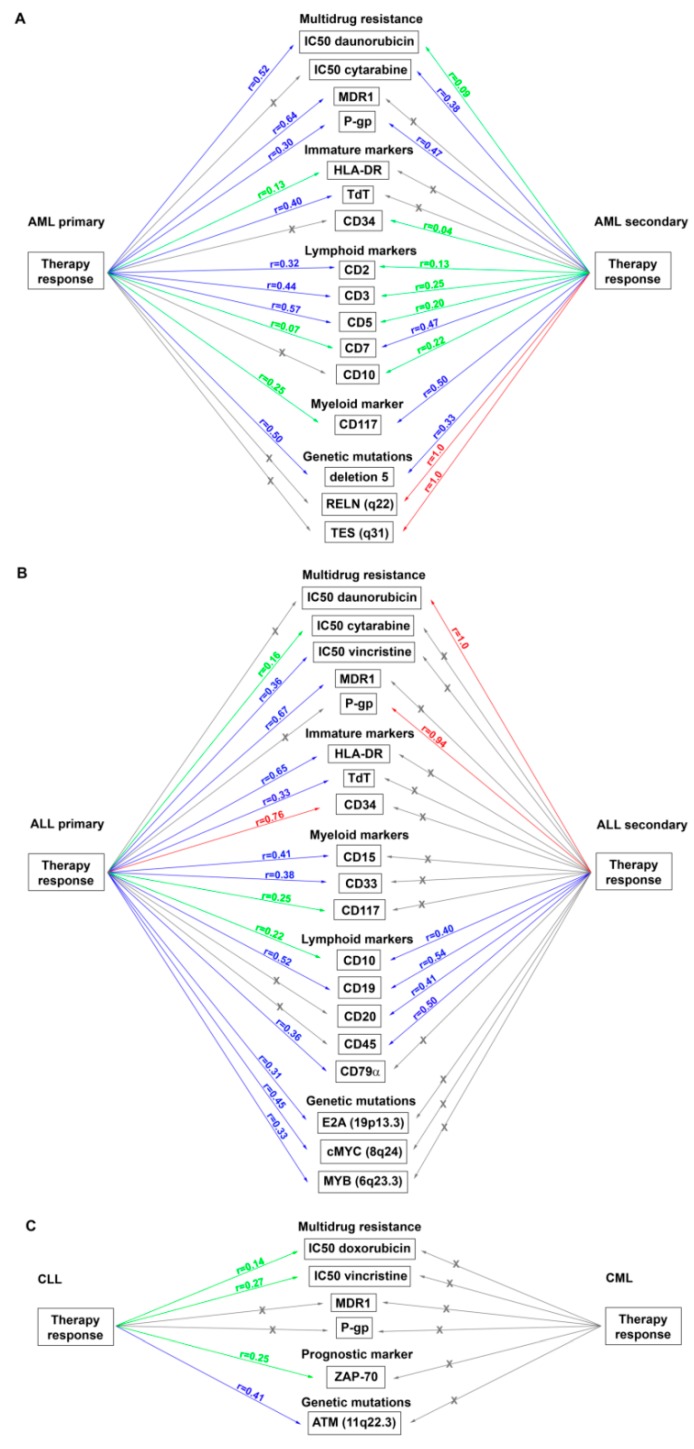
Correlations between the therapy response, cell sensitivity to chemotherapeutic drugs, *MDR1* mRNA and P-glycoprotein expression, immunological markers, and cytogenetic abnormalities in leukemia patients. Correlation coefficients reflecting the statistical relationships between the studied parameters were evaluated for patients with acute myeloid leukemia (**A**), acute lymphoblastic leukemia (**B**), chronic lymphocytic leukemia and chronic myeloid leukemia (**C**). Red arrows indicate strong positive correlations (0.70 ≤ r_s_ ≤ 1.00), blue arrows indicate moderate positive correlations (0.30 ≤r_s_ ≤ 0.69), green arrows indicate weak positive correlations (0.01 ≤ r_s_ ≤ 0.29), and gray arrows indicate no correlations.

For patients with primary AML, moderate correlations between the therapy response and sensitivity of tumor cells to daunorubicin (*r* = 0.52), but not to doxorubicin (*r* = −0.25) and cytarabine (*r* = −0.06), were found (Figure 3A). As expected in this cohort, moderate correlations between the therapy response and expression in tumor cells of a number of immunological markers of T-lymphocytes, which reflects an aberrant immunophenotype for leukemia with myeloid origin and may be associated with unfavorable outcomes [39,40], were found: CD2 (*r* = 0.32), CD3 (*r* = 0.44), and CD5 (*r* = 0.57) (Figure 3A). Statistically significant relationships between the therapy response and immunological markers of cell immaturity, an expression of which is associated with poor prognosis [39,41] (HLA-DR, *r* = 0.13 and TdT, *r* = 0.40), were also found (Figure 3A). Thus, the blast cells of patients with primary AML, who did not respond to treatment, demonstrated low sensitivity or resistance to chemotherapeutic drugs in vitro. These patients exhibited an aberrant immunophenotype and immature markers related to unfavorable prognosis and an aggressive course of the disease. Also, it should be mentioned that the blast cells of patients who responded to treatment and achieved remission, exhibited high drug sensitivity and did not express unfavorable markers.

Next, we separately analyzed the relationships between the drug sensitivity of tumor cells of patients with primary AML and the expression of lymphoid (T- and B-) and immature markers as well as the proliferation marker, Ki-67, which are indicators of an unfavorable prognosis for leukemia [39,40,41,42]. Resistance of tumor cells to doxorubicin correlated only with the expression of one T-cell marker, CD2 (*r* = 0.51), whereas resistance to daunorubicin correlated directly with the expression of several T-cell markers (CD2, *r* = 0.16; CD3, *r* = 0.38; CD7, *r* = 0.07; and CD11c, *r* = 0.41), and the natural killer (NK) cell marker, CD56 (*r* = 0.50). Similar correlations were found for cytarabine: CD3 (*r* = 0.23), CD7 (*r* = 0.05), CD11c (*r* = 0.97), and CD56 (*r* = 0.17) (Table S5). Interestingly, expression of the proliferation marker, Ki-67, correlated only with resistance to daunorubicin (*r* = 0.35), but not with other chemotherapeutics. Similarly, only weak correlations between the immaturity markers, HLA-DR and TdT, and resistance to daunorubicin and cytarabine were found (Table S5).

An evaluation of the relationships between cytogenetic abnormalities in tumor cells, therapy response, and cell sensitivity or resistance to chemotherapeutic drugs in patients with primary AML revealed a moderate correlation between the therapy response and deletion 5 (*r* = 0.50), which has the worse prognostic impact in combination with some other genetic mutations [43], and a strong correlation between the resistance to cytarabine and mutation of P53(17p13.1) (*r* = 0.94), responsible for the impairment of P53 tumor suppressor pathways [44] (Figure 3A, Table S6).

These data confirm our assumption that the sensitivity of tumor cells estimated in vitro closely correlates with the response of leukemia patients to antitumor therapy. It should also be noted that, for example, resistance to daunorubicin has a more prognostic significance than other cytotoxic drugs in patients with primarily diagnosed AML.

#### 3.4.2. Secondary AML

In patients with secondary AML, the therapy response correlated with the sensitivity of tumor cells to cytarabine (*r* = 0.38) and daunorubicin (*r* = 0.09), but not with doxorubicin (*r* = −0.16) (Figure 3A). In this cohort, moderate relationships between the resistance of tumor cells to doxorubicin and expression of T-cell markers (CD2, *r* = 0.42 and CD16, *r* = 0.50) were found (Table S5). At the same time, resistance to daunorubicin correlated with a significantly greater number of T-cell markers (CD2, *r* = 0.48; CD3, *r* = 0.45; CD7, *r* = 0.38; CD5, *r* = 0.34; and CD16, *r* = 0.34). Moreover, for daunorubicin, but not for doxorubicin, correlations with unfavorable predictive markers were found: Immaturity markers (HLA-DR, *r* = 0.05 and CD34, *r* = 0.24) and the proliferation marker, Ki-67 (*r* = 0.87) (Table S5). Resistance of tumor cells to cytarabine correlated with the expression of Ki-67 (*r* = 0.33) and T-cell markers (CD2, *r* = 0.22; CD3, *r* = 0.25; and CD5, *r* = 0.14), but not with immaturity markers (Table S5).

In assessing the cytogenetic abnormalities in leukemic cells of patients with secondary AML, a strong correlation between the therapy response and mutations associated with primary chemoresistance, RELN(q22) [45], and alteration of tumor suppression, TES(q31) [46], was detected (*r* = 1.0 for both mutations) (Figure 3A). There was also a moderate correlation between the response to treatment and deletion 5 (Figure 3A). Correlations between the RELN(q22) and TES(q31) mutations and drug resistance of leukemic cells were found: Moderate correlations with daunorubicin (*r* = 0.33 for both mutations) and weak correlations with cytarabine (*r* = 0.27 for both mutations) (Table S6). A strong correlation of translocation t(9;22)(q34;q11), which predicted a significantly poorer outcome in AML patients [47], with resistance to daunorubicin (*r* = 0.71) and a moderate correlation with resistance to cytarabine (*r* = 0.58) were also shown. Finally, moderate correlations of resistance to cytarabine and cytogenetic abnormalities, associated with poor prognosis, including P53(17p13.1) and EVI1(3q26.2) [48], were revealed (*r* = 0.58 and *r* = 0.50, respectively) (Table S6).

Thus, the correlations identified for secondary AML coincide with the data for primary AML to a certain extent: Evaluation of the sensitivity or resistance of tumor cells to anthracycline drugs (daunorubicin and, to a lesser extent, doxorubicin) is of greater prognostic significance for the assessment and prediction of the patient’s response to antitumor therapy. However, for patients with secondary AML, sensitivity to cytarabine was found to be of a certain prognostic value compared with primary AML patients.

#### 3.4.3. Primary ALL

In patients with primary ALL, moderate and weak correlations between the therapy response and the sensitivity of tumor cells to vincristine (*r* = 0.36) and cytarabine (*r* = 0.16) were found (Figure 3B). Statistical relationships between the therapy response and the sensitivity to anthracyclines were not revealed. Also, in this cohort, correlations between the therapy response and the expression in tumor cells of myeloid markers, the presence of which reflects an aberrant immunophenotype for leukemia with lymphoid origins [39,40], were found: Moderate correlations with CD33 (*r* = 0.38) and CD15 (*r* = 0.41); and weak correlations with CD117 (*r* = 0.25) and CD65 (*r* = 0.29) (Figure 3B). Correlations between the therapy response and the immaturity markers, which are the most useful ways of detecting minimal residual disease and predicting relapse in ALL [49], were also found: A strong correlation with CD34 (*r* = 0.76) and a moderate correlation with HLA-DR (*r* = 0.65) and TdT (*r* = 0.33).

An evaluation of relationships between the drug sensitivity of tumor cells from patients with primary ALL and the expression of immunological markers showed moderate correlations between the expression of the myeloid marker, CD33, and resistance to doxorubicin (*r* = 0.59), vincristine (*r* = 0.53), and cytarabine (*r* = 0.62) (Table S7). Relationships were also found between the resistance to vincristine and immaturity markers (HLA-DR, *r* = 0.50 and CD34, *r* = 0.37) (Table S7).

There were moderate correlations between the therapy response of patients with primary ALL and the presence of cytogenetic abnormalities, which correlate with tumorigenesis and the MDR status of leukemia patients [50,51,52]: E2A(19p13.3) (*r* = 0.31), cMYC(8q24) (*r* = 0.45), and MYB(6q23.3) (*r* = 0.33) (Figure 3B). Also, the MLL(11q23.3) mutation in charge of dismal prognosis and common therapy refractoriness in ALL patients [53] correlated with the resistance to all studied antitumor drugs: Daunorubicin (*r* = 0.33), cytarabine (*r* = 0.32), doxorubicin (*r* = 0.29), and vincristine (*r* = 0.22) (Table S6). Correlations between the translocation, t(9;22)(q34;q11), which is one of the most frequent cytogenetic abnormalities in ALL patients [54], and resistance to doxorubicin (*r* = 0.55), vincristine (*r* = 0.26), daunorubicin (*r* = 0.09), and cytarabine (*r* = 0.09) were also detected. Mutation E2A(19p13.3) exhibited moderate correlations with resistance to doxorubicin and cytarabine (*r* = 0.45 and *r* = 0.54, respectively) and a weak correlation with resistance to daunorubicin (*r* = 0.20) (Table S6). Also noteworthy is the fact that in patients with primary ALL, the resistance of leukemic cells to cytarabine correlated with the presence of almost all diagnostic genetic anomalies: MLL(11q23.3) (*r* = 0.32), translocation t(9;22)(q34) (*r* = 0.09), E2A(19p13.3) (*r* = 0.54), cMYC(8q24) (*r* = 0.61), and MYB(6q23.3) (*r* = 0.50) (Table S6).

Thus, tumor cells of primary ALL patients with poor therapy response are characterized by a low sensitivity or resistance to antitumor drugs in vitro, expression of aberrant and immaturity markers, and the presence of unfavorable genetic mutations; however, patients who responded to antitumor treatment and achieved remission had high drug sensitivity and did not exhibit markers of unfavorable prognosis.

#### 3.4.4. Secondary ALL

In patients with secondary ALL, significant correlations between the therapy response, the sensitivity of tumor cells to daunorubicin (*r* = 1.0), and the expression of the progenitor marker, CD45 (*r* = 0.50), which is one of the stratification markers of ALL [55], were found (Figure 3B). Also, in this cohort, a moderate correlation between the expression of the T-cell marker, CD2, which sometimes has prognostic significance, and resistance to doxorubicin (*r* = 0.50), vincristine (*r* = 0.50), and cytarabine (*r* = 0.33) were also revealed (Table S7). The immaturity marker, CD34, was found to be moderately correlated with resistance to doxorubicin (*r* = 0.50) and vincristine (*r* = 0.50), and strongly correlated with the resistance to cytarabine (*r* = 1.0) (Table S7).

We were not able to perform a correlation analysis when assessing the cytogenetic abnormalities of leukemia cells of patients in this cohort due to the small sample size.

#### 3.4.5. CLL

In patients with CLL, correlations between the therapy response and the sensitivity of tumor cells to doxorubicin (*r* = 0.14) and vincristine (*r* = 0.27) were found (Figure 3C). There were no statistically significant correlations between the therapy response and the leukocyte level in the PB and lymphocyte level in the BM, which are parameters for the staging of CLL [56]. However, correlations between the therapy response and the expression of immunological and genetic markers, which play a prognostic role in CLL and predict short treatment free survival and poor outcome, including ZAP-70 [57] and ATM(11q22.3) [58], were detected (*r* = 0.25 and *r* = 0.41, respectively) (Figure 3C).

In CLL patients, weak and moderate correlations between the cell resistance to doxorubicin and the expression of the unfavorable prognostic marker, CD38 [59] (*r* = 0.23); the proliferation marker, Ki-67 (*r* = 0.12); and the immaturity marker, HLA-DR (*r* = 0.43), were found (Table S8). In addition, correlations between the resistance of tumor cells to vincristine and the expression of Ki-67 (*r* = 0.13) and ZAP-70 (*r* = 0.61) were detected (Table S8). There were no correlations between the drug sensitivity of tumor cells of this group of patients and the presence of cytogenetic abnormalities.

#### 3.4.6. CML

In patients with CML, no correlations between the therapy response and the sensitivity of tumor cells to cytotoxic drugs were found (Figure 3C). This may be due to the small sample size as well as the prolonged course of the disease. Correlations between the cytogenetic abnormalities, such as trisomies (chromosomes 6, 7, 8, 17, and 21), and resistance to daunorubicin (*r* = 0.58) and cytarabine (*r* = 0.24) were detected (Table S6).

### 3.5. Correlations of MDR1 mRNA and P-Glycoprotein Expression in Tumor Cells with Drug Sensitivity and Therapy Response in Patients with Acute and Chronic Leukemia

Next, we studied the relationships between the sensitivity of leukemia cells to cytotoxic drugs detected in vitro and the MDR phenotype mediated by P-gp—a product of the *MDR1* gene. The primary data on *MDR1* mRNA and P-gp expression levels for each patient are presented in Figure 1 and Figure 2. In primary AML patients, high expression of *MDR1* mRNA exhibited moderate correlations with poor therapy response (*r* = 0.64) and resistance to cytarabine (*r* = 0.53), and strong correlations with resistance to daunorubicin (*r* = 0.74) (Figure 3A, Table S9). For secondary AML patients, high expression of *MDR1* mRNA correlated only with the resistance to daunorubicin (*r* = 0.44) (Figure 3A, Table S9). In patients with primary ALL, moderate correlations between *MDR1* mRNA expression and therapy response (*r* = 0.67) and weak correlations between the same parameter and sensitivity to daunorubicin, cytarabine, and vincristine (*r* = 0.14, *r* = 0.09, and *r* = 0.10, respectively) were found (Figure 3B, Table S9). Patients with secondary ALL were not included in this part of the study because of the small sample size. Patients with CLL had statistically significant correlations of *MDR1* mRNA expression only with sensitivity to vincristine (*r* = 0.25) (Figure 3C, Table S9). Thus, an evaluation of drug resistance in tumor cells of leukemia patients measured as *MDR1* mRNA levels has prognostic value for individual hematological disorders, in particular for newly diagnosed patients with AML.

Patients with primary AML had moderate correlations between P-gp expression and therapy response (*r* = 0.30) and no correlations between P-gp expression and drug sensitivity (Figure 3A, Table S9). Patients with secondary AML had significant correlations between P-gp expression, therapy response (*r* = 0.47), and sensitivity to daunorubicin (*r* = 0.62) and cytarabine (*r* = 0.25) (Figure 3A, Table S9). Patients with primary ALL had no correlations between P-gp expression, therapy response, and sensitivity to anthracyclines, and had weak correlations between high expression of P-gp and resistance to cytarabine (*r* = 0.15) and vincristine (*r* = 0.06) (Figure 3B, Table S9). Patients with secondary ALL had strong correlations between P-gp expression and therapy response (*r* = 0.94) and had no correlations between P-gp expression and drug sensitivity (Figure 3B, Table S9). No correlations between P-gp expression, therapy response, and cell sensitivity to chemotherapeutic drugs in CLL patients were detected (Figure 3C, Table S9).

Thus, it can be assumed that the sensitivity or resistance of tumor cells to cytotoxic drugs detected in vitro correlates with the indicators of one of the main resistance mechanisms—*MDR1* mRNA and P-gp expression levels—and has prognostic value for acute leukemias. The evaluated level of *MDR1* mRNA has a greater importance at the initial diagnosis, and P-gp for pre-treated leukemia patients.

## 4. Discussion

Multidrug resistance (MDR) is defined as the insensitivity of cancer cells to chemotherapeutic drugs with different mechanisms of action and toxic profiles, which develops after one or more treatment courses. The treatment of different cancers carrying MDR is a major challenge, and rates of morbidity and mortality are high [60,61].

The resistance of tumor cells to chemotherapeutic agents especially in leukemia patients, regardless of the reasons and mechanisms of its development, complicates treatment, makes therapy ineffective, and worsens the prognosis of the disease [62,63]. The significant problems in leukemia treatment are the variability in the maturation state of leukemic cells at origin, multiformity of genetic aberrations among patients, and the existence of multiple disease clones within a single patient [64]. Disease heterogeneity and a lack of biomarkers for drug sensitivity or resistance lie at the heart of the selective efficacy of chemotherapies, emergence of drug resistance, and treatment failure of leukemia patients [65]. It has also been shown in clinical practice that the emergence of MDR causes considerable levels of pain in many leukemia patients and a significant decrease in quality of life [66]. Therefore, the early, accurate, and selective detection of the MDR of tumor cells is vital, and is also beneficial in the search for more effective chemotherapeutic approaches for use in the clinical setting.

The aim of our study was to search for instruments allowing for rapid and economical diagnosis of drug sensitivity or resistance of tumor cells to assess antitumor therapy possibilities for each patient without the need to conduct multiple complicated analyses. We assumed that the administration of chemotherapeutics to leukemia patients should be conducted according to the drug responsiveness of tumor cells. Our study included 113 hematological patients with leukemias. We evaluated the sensitivity of tumor cells (IC_50_ values) for a panel of chemotherapeutic drugs using the WST-test as well as *MDR1* mRNA and P-gp levels as one of the possible mechanisms of drug resistance for leukemia patients prior to the start of cytotoxic therapy. The scales for distribution of leukemia patients according to the therapy response, drug sensitivity or resistance of tumor cells, *MDR1* mRNA and P-gp expression levels, and the presence or absence of prognostic immunological and genetic markers were developed for subsequent correlation analysis.

For acute leukemia, many important correlations between the therapy response, drug sensitivity of tumor cells, and key immunological and cytogenetic markers were found (Figure 3A,B), opposite to chronic leukemia, for which significantly fewer correlations were identified (Figure 3C). We also found that the drug resistance of leukemic cells detected in vitro correlates more significantly with the therapy response and immunological and genetic markers than *MDR1* mRNA and P-gp levels. It is well known that there are numerous mechanisms of MDR formation, which can be activated simultaneously in tumor cells [7,8]. The WST-test evaluates the total drug responsiveness (sensitivity or resistance) of tumor cells regardless of the mechanism, whereas data on *MDR1* mRNA and P-gp expression indicate only MDR1/P-gp-dependent MDR.

At present, methods commonly used for gene detection involved in MDR formation include PCR, in situ hybridization, and RNase protection assays. Western blotting and immunohistochemistry may also be used for protein detection [7]. However, these techniques take more time and are more labor-intensive and expensive, so they are less suitable for screening and can be used in clinical practice mainly to clarify the MDR mechanism and for certain groups of leukemia patients. For example, in our study, *MDR1* mRNA evaluation had a stronger prognostic value for patients with primary acute leukemia, whereas P-gp expression levels more strongly correlated with the therapy response, drug sensitivity, and prognostic markers in patients with secondary acute leukemia. Thus, it can be assumed that it is preferable to use enzyme-based methods (MTT, WST, etc.) to assess cell viability in vitro to screen the sensitivity of tumor cells to cytotoxic drugs and predict the therapy response. It should be noted that the methods mentioned above could be combined for more accurate prediction of therapy efficiency in leukemia patients [67]. In our study, we found that the evaluation of drug sensitivity or resistance of tumor cells of leukemia patients in vitro by the WST-test has a different prognostic value for different types of leukemia. Moreover, we showed that this diagnostic approach can be effectively used only for particular types of leukemia, especially for AML. However, in regard to chronic leukemias, it does not have a significant prognostic value, and other methods of predicting the responsiveness to chemotherapy and treatment efficacy should be used for this group of hematological diseases.

**Figure 4:**
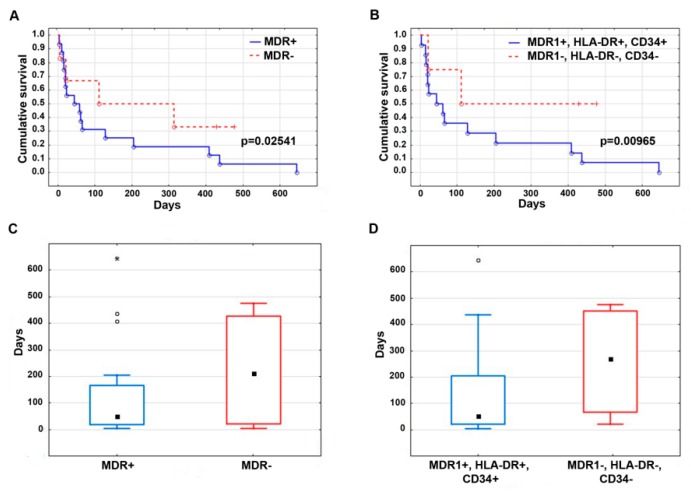
Survival of leukemia patients depending on the presence or absence of unfavorable prognosis factors. The Kaplan–Meier survival curves (**A**,**B**) and median follow-up boxes (**C**,**D**) were plotted for acute myeloid leukemia (AML) patients with or without *MDR1* mRNA expression (MDR1+ and MDR1−, respectively) (**A**,**C**) and with or without simultaneous expression of *MDR1* mRNA, HLA-DR, and CD34 (MDR1+, HLA-DR+, CD34+ and MDR1−, HLA-DR−, CD34−, respectively) (**B**,**D**).

We also evaluated the survival of leukemia patients, depending on the presence or absence of unfavorable prognostic factors. Figure 4 demonstrates the Kaplan–Meier curves (Figure 4A,B) and median follow-up (Figure 4C,D) of AML patients with the presence or absence of the immunological markers, HLA-DR and CD34, as well as *MDR1* mRNA expression. Median follow-up was 135.1 days in AML patients with positive expression of *MDR1* mRNA and 225.8 days with negative expression of *MDR1* mRNA in leukemic cells. The median follow-up was 149.4 days in AML patients with simultaneous positive expression of *MDR1* mRNA, CD34, and HLA-DR, and 259.3 days without expression of one of the studied markers. Statistically significant increased survival of AML patients was observed in the absence of the *MDR1* mRNA expression and/or the immature markers, HLA-DR and CD34, in blast cells. The obtained data on patient survival fit well with the information available in public databases (Platform: SurvExpress; Database: Metzeler Buske CM-AML GSE12417-GPL570). There was also a higher survival rate of AML patients with a high sensitivity of tumor cells to daunorubicin and cytarabine, although these differences were not statistically significant.

Thus, we believe that administration of chemotherapeutic drugs individually according to the cell sensitivity evaluated in vitro can provide more favorable treatment results, reduce the risk of relapse, and ultimately, improve the prognosis of patients with malignant diseases.

## 5. Conclusions

The development of MDR is one of the major challenges in the success of traditional chemotherapy in leukemia patients. Many studies have focused on strategies to reverse MDR following its development. However, agents utilizing this approach have limited clinical use and demonstrate a failure of achieving improvements in therapeutic efficacy, with almost no significant survival benefits [68]. An alternative approach that has been applied is to prevent or delay MDR. Recent investigations have shown that preventing the emergence of MDR at the onset of chemotherapy treatment can be achieved by a rational approach to the administration of cytotoxic drugs in leukemia patients [69].

In this study, we have shown that the drug sensitivity of tumor cells of leukemia patients, estimated in vitro, correlates with the therapy response and markers of unfavorable prognosis and has a significant predictive value for individual leukemia types, in particular for acute leukemias. In regard to chronic leukemias, it does not have a significant prognostic value, and other methods predicting the responsiveness to chemotherapy should be used.

A comprehensive approach using drug sensitivity tests at diagnosis, which can be expanded by the evaluation of MDR gene expression, allows clinical individualization of chemotherapy, which will be one of the key steps in the development of personalized medicine for leukemia patients.

## Figures and Tables

**Table 1:** Scales for the distribution of leukemia patients according to the sensitivity of tumor cells to chemotherapeutic drugs.

Chemotherapeutic Drug	IC_50_ Values (Number of Patients)		
	1—High Sensitivity	2—Moderate Sensitivity	3—Low Sensitivity (Resistance)
	AML		
**Doxorubicin, µM**	0–0.5 (8)	0.5–1 (0)	>1 (1)
**Daunorubicin, µM**	0–0.25 (17)	0.25–0.5 (10)	>0.5 (15)
**Cytarabine, µM**	0–1.5 (9)	1.5–8 (9)	>8 (28)
	ALL		
**Doxorubicin, µM**	0–0.05 (5)	0.05–0.1 (0)	>0.1 (8)
**Daunorubicin, µM**	0–0.1 (5)	0.1–0.25 (2)	>0.25 (8)
**Cytarabine, µM**	0–5 (4)	5–10 (0)	>10 (9)
**Vincristine, µM**	0–0.01 (3)	0.01–0.015 (1)	>0.015 (11)
	CLL		
**Doxorubicin, µM**	0–0.25 (15)	0.25–0.5 (8)	>0.5 (15)
**Vincristine, µM**	0–0.05 (13)	0.05–0.1 (6)	>0.1 (19)
	CML		
**Doxorubicin, µM**	0–0.25 (0)	0.25–0.5 (1)	>0.5 (2)
**Daunorubicin, µM**	0–0.5 (2)	0.5–1 (0)	>1 (1)
**Cytarabine, µM**	0–50 (1)	50–100 (1)	>100 (3)

The number of patients in each group is indicated in parentheses.

## Supplementary Materials

The following are available online at https://www.mdpi.com/2075-4426/9/2/24/s1, Table S1: Monoclonal antibodies used for immunophenotyping of leukemia patients by flow cytometry, Table S2: Total number, average age and gender distribution of leukemia patients included in the study, Table S3: Sensitivity of tumor cells to chemotherapeutic drugs (WST-test data), treatment regimens and therapy response of patients with acute and chronic leukemia with scales for correlation analysis (in parentheses), Table S4: Immunological markers and cytogenetic abnormalities detected in tumor cells of patients with acute and chronic leukemia with scales for correlation analysis (in parentheses), Table S5: Correlation coefficients reflecting the relationships between the drug sensitivity and expression of immunological markers in tumor cells of patients with acute myeloid leukemia, Table S6: Correlation coefficients reflecting the relationships between the drug sensitivity and cytogenetic abnormalities in tumor cells of leukemia patients, Table S7: Correlation coefficients reflecting the relationships between the drug sensitivity and expression of immunological markers in tumor cells of patients with acute lymphoblastic leukemia, Table S8: Correlation coefficients reflecting the relationships between the drug sensitivity and expression of immunological markers in tumor cells of patients with chronic lymphocytic leukemia, Table S9: Correlation coefficients reflecting the relationships between the drug sensitivity, *MDR1* mRNA, and P-glycoprotein expression in tumor cells of leukemia patients.
