# Supplementary material for: Clinical and Prognostic Significance of Cell Sensitivity to Chemotherapy Detected In Vitro on Treatment Response and Survival of Leukemia Patients
Source: J Pers Med. 2019 May 7;9(2):24. doi: 10.3390/jpm9020024 (PMC6617197; doi:10.3390/jpm9020024)
Supplement: Supplementary file 1 [file jpm-09-00024-s001.pdf]

Journal of *Personalized Medicine*

MDPI

Supplementary Materials

Table S1. Monoclonal antibodies used for immunophenotyping of leukemia patients by flow cytometry.

Table S2. Total number, average age and gender distribution of leukemia patients included in the study.

Table S3. Sensitivity of tumor cells to chemotherapeutic drugs (WST-test data), treatment regimens and therapy response of patients with acute and chronic leukemia with scales for correlation analysis (in parentheses).

Table S4. Immunological markers and cytogenetic abnormalities detected in tumor cells of patients with acute and chronic leukemia with scales for correlation analysis (in parentheses).

Table S5. Correlation coefficients reflecting the relationships between the drug sensitivity and expression of immunological markers in tumor cells of patients with acute myeloid leukemia.

Table S6. Correlation coefficients reflecting the relationships between the drug sensitivity and cytogenetic abnormalities in tumor cells of leukemia patients.

Table S7. Correlation coefficients reflecting the relationships between the drug sensitivity and expression of immunological markers in tumor cells of patients with acute lymphoblastic leukemia.

Table S8. Correlation coefficients reflecting the relationships between the drug sensitivity and expression of immunological markers in tumor cells of patients with chronic lymphocytic leukemia.

Table S9. Correlation coefficients reflecting the relationships between the drug sensitivity, *MDR1* mRNA and P-glycoprotein expression in tumor cells of leukemia patients.

Table 1. Monoclonal antibodies used for immunophenotyping of leukemia patients by flow cytometry.

Immunological marker	Catalogue number	Manufacture company
CD16-FITC/CD56-PE/CD3-ECD	A07728	Beckman Coulter, USA
CD19-PC5	A07771	Beckman Coulter, USA
CD20-FITC/CD10-PE/CD19-ECD	A07708	Beckman Coulter, USA
CD34-PC5	A07777	Beckman Coulter, USA
CD117-PE	IM2732	Beckman Coulter, USA
CD5-PC5	A07754	Beckman Coulter, USA
CD34-FITC	IM1870	Beckman Coulter, USA
CD20-PC5	A07773	Beckman Coulter, USA
CD7-PC5	IM3613	Beckman Coulter, USA
HLA-DR-FITC/CD13-PE/CD45-ECD	A07720	Beckman Coulter, USA
CD65-FITC	IM1645U	Beckman Coulter, USA
CD10-PC5	A07761	Beckman Coulter, USA

CD38-PC5	A07780	Beckman Coulter, USA
CD38-FITC	A07778	Beckman Coulter, USA
CD20-PC7	IM3629	Beckman Coulter, USA
CD19-PC7	IM3628	Beckman Coulter, USA
CD4-FITC	A07750	Beckman Coulter, USA
CD25-PC5	IM2646	Beckman Coulter, USA
CD7-PC5	IM3613	Beckman Coulter, USA
CD19-ECD	A07770	Beckman Coulter, USA
CD5-FITC/CD23-PE/CD19-ECD	A07710	Beckman Coulter, USA
CD22-PE	IM1835	Beckman Coulter, USA
CD45-PC7	IM3548	Beckman Coulter, USA
CD103-FITC/CD11c-PE/CD19-ECD	A07712	Beckman Coulter, USA
Anti-HLA-DR-PE	IM1639	Beckman Coulter, USA
CD56-PC5	A07789	Beckman Coulter, USA
Anti-TCR-PANγ/δ-PC5	IM2662	Beckman Coulter, USA
CD3-ECD	A07748	Beckman Coulter, USA
CD2-PC5	A07745	Beckman Coulter, USA
CD5-FITC/CD7-PE/CD3-ECD	A07725	Beckman Coulter, USA
CD138-PE	A54190	Beckman Coulter, USA
CD16-PE	A07766	Beckman Coulter, USA
CD117-PC5	IM2733	Beckman Coulter, USA
FMC7-FITC	A07791	Beckman Coulter, USA
CD45-ECD	A07784	Beckman Coulter, USA
Anti-TCR-α/β(WT31)	333140	BD Biosciences, USA
CD26(L272)-FITC	340426	BD Biosciences, USA
CD26(L272)-PE	340423	BD Biosciences, USA
TdT (Pool)-FITC	IM3524	Beckman Coulter, USA

cMPO-FITC/cCD79a-PE/cCD3-ECD	A07705	Beckman Coulter, USA
Kappa-FITC/Lambda-PE/CD19-ECD	A07706	Beckman Coulter, USA
CD30-PE	333906	BioLegend, USA
Ki67-PE	350504	BioLegend, USA
CD61-FITC	IM1758	Beckman Coulter, USA
CD22-PE	IM1835	Beckman Coulter, USA
CD13-PE	A07762	Beckman Coulter, USA
CD15-FITC/CD33-PE/CD45-ECD	A07721	Beckman Coulter, USA
CD45-FITC	A07782	Beckman Coulter, USA
CD45-FITC/Glycophorin A (CD235a)-PE/CD45-ECD	A07723	Beckman Coulter, USA
CD45-FITC CD14-PE	A07738	Beckman Coulter, USA
CD45-PC5	A07785	Beckman Coulter, USA
CD3-PC5	A07749	Beckman Coulter, USA
CD11c-PC5	IM3707	Beckman Coulter, USA
CD71-FITC/CD33-PE/CD45-ECD	A07716	Beckman Coulter, USA
cMPO-FITC/cCD79a-PE/cCD3-ECD	A07705	Beckman Coulter, USA
CD25-FITC/CD11c-PE/CD19-ECD	A07711	Beckman Coulter, USA
CD64-PC5	IM3606U	Beckman Coulter, USA
CD43-FITC	IM3264U	Beckman Coulter, USA
CD68-FITC	MCA5709F	Bio-Rad, USA

Table 2. Total number, average age and gender distribution of leukemia patients included in the study.

	In total	Average age, years	Gender distribution	
			Men	Women
**AML**	49	49.8±13.5	46.9% (23)	53.1% (26)
**ALL**	16	42±16.2	68.8% (11)	31.2% (5)
**CLL**	43	64±8.6	62.8% (27)	37.2% (16)
**CML**	5	46±14.5	60% (3)	40% (2)

Table 3. Sensitivity of tumor cells to chemotherapeutic drugs (WST-test data), treatment regimens and therapy response of patients with acute and chronic leukemia with scales for correlation analysis (in parentheses).

Patient#	Diagnosis	IC$_{50}$, M (scale)				Treatment regimens	Therapy response (scale)
		Doxorubicin	Daunorubicin	Cytarabine	Vincristine		
				**AML (primary)**			
**1**	**AML**		0.4±0.05 (2)	3.6±1.0 (2)		no	remission (1)
2	AML		0.08±0.01 (3)	60±21.6 (3)		AIDA	remission (1)
3	AML		0.4±0.05 (2)			7+3; 7+3+VP; 5+2	remission(1)
4	AML		0.4±0.25 (2)	2.9±1.2 (2)		no	relapse (2)
5	AML		0.06±0.01 (1)	0.3±0.25 (2)		6-MP	remission(1)
6	AML		0.2±0.02 (1)	2.5±0.8 (2)		6-MP	relapse (2)
7	AML	>2 (3)	>2 (3)	>82 (3)		low-dose cytarabine-21	resistance (3)
8	AML	0.2±0.05 (1)	0.05±0.01 (1)	0.7±0.3 (1)		no	remission (1)
9	AML		>8 (3)	>82 (3)		7+3; HAM; 6-MP	resistance (3)
10	AML		0.2±0.03 (2)	22.8±3.7 (3)		7+3; high-dose cyclophosphamide; HAM	remission (1)
11	AML		0.2±0.05 (1)	0.6±0.3 (1)		7+3; HiDAC; 6-MP	resistance (3)
12	AML		>2 (3)	>82 (3)		no	resistance (3)
13	AML		0.2±0.03 (1)	>82 (3)		7+3; low-dose cytarabine-21	remission (1)
14	AML	0.1±0.01 (1)	0.01±0.001 (1)	0.3±0.1 (1)		AIDA	remission(1)
15	AML	0.5±0.3 (1)	0.3±0.05 (2)	64.4±27 (3)		7+3	relapse (2)
16	AML	0.35±0.1 (1)	0.2±0.02 (1)	2.9±0.7(2)		low-dose cytarabine-21+6-MP	remission (1)
17	AML		0.1±0.01 (1)	14.8±2 (3)		6-MP	relapse (2)
18	AML	0.4±0.1 (1)	0.02±0.001 (1)	2.3±1.3 (2)		7+3; low dose cytarabine-14; 5+2; intermediate-dose cytarabine-7	relapse (2)
19	AML		0.02±0.002 (1)	0.6±0.4 (1)		AIDA	remission (1)
20	AML		0.2±0.01 (1)	0.9±0.2 (1)		7+3	relapse (2)
21	AML	0.4±0.06 (1)		0.7±0.2 (1)		7+3+vesanoid; 6-MP	remission (1)
22	AML		>3.5 (3)	>40.8 (3)		7+3; HiDAC; low-dose cytarabine-21; 6-MP	resistance (3)
23	AML		>8 (3)	>82 (3)		low dose cytarabine-14; 6-MP	resistance (3)
24	AML		1.3±0.4 (3)	>82 (3)		7+3; low-dose cytarabine-21	resistance (3)

25	AML		>2 (3)	>82 (3)	7+3	resistance (3)
26	AML		8.8±0.02 (3)	0.3±0.1 (1)	no	remission (1)
27	AML	0.02±0.002 (1)	0.01±0.002 (1)	0.9±0.4 (1)	low-dose cytarabine-21	resistance (3)
28	AML		0.4±0.3 (2)	16.9±5.5 (3)	low-dose cytarabine-21; 6-MP	resistance (3)
29	AML		0.3±0.02 (2)	71.5±21 (3)	low-dose cytarabine-21; 5+2	resistance (3)
30	AML	0.5±0.01 (1)		22±11.2 (3)	7+3	remission (1)
			**AML (secondary)**			
31	AML		>2 (3)	>82 (3)	5+2; 7+3; HAM; intermediate-dose cytarabine-7	resistance (3)
32	AML	>2 (3)	>2 (3)	>82 (3)	low-dose cytarabine-14; 5+2	resistance (3)
33	AML	0.2±0.035 (1)			low-dose cytarabine-21+dasatinib	relapse (2)
34	AML	0.3±0.05 (1)	0.2±0.035 (1)	1.1±0.3 (1)	low-dose cytarabine-28; 5+2; 6-MP	resistance (3)
35	AML	0.4±0.04 (1)	0.35±0.08 (2)	3±0.6 (2)	5+2; 7+3; low-dose cytarabine-14	relapse (2)
36	AML		>8 (3)	>82 (3)	5+2; low-dose cytarabine-28; 7+3; VAD; 6-MP	resistance (3)
37	AML	3±0.6 (3)	0.45±0.07 (2)	123.4±11 (3)	no	resistance (3)
38	AML	>2 (3)	>2 (3)	>82 (3)	low-dose cytarabine-21; low-dose cytarabine-14	resistance (3)
39	AML		0.2±0.02 (1)	29±5 (3)	low-dose cytarabine-21	resistance (3)
40	AML	>2 (3)		>82 (3)	5+2	resistance (3)
41	AML			3.6±0.2 (2)	7+3; AIDA; intermediate-dose cytarabine-7; 6-MP	relapse (2)
42	AML	>2 (3)	4.4±1.5 (3)	>82 (3)	low-dose cytarabine-21+dasatinib	remission (1)
43	AML		0.2±0.01 (1)	2±0.5 (2)	low-dose cytarabine-21	relapse (2)
44	AML	0.7±0.1 (2)	0.5±0.1 (2)	>82 (3)	low-dose cytarabine-21; 6-MP	resistance (3)
45	AML	0.3±0.03 (1)			no	resistance (3)
46	AML		0.2±0.02 (1)	>82 (3)	no	resistance (3)
47	AML	0.3±0.04 (1)	0.2±0.01 (1)	13.6±3.3 (3)	low-dose cytarabine-28; 6-MP	resistance (3)
48	AML	>2 (3)		>161 (3)	low-dose cytarabine-14	relapse (2)

49	AML		>2 (3)	>82 (3)		5+2; low-dose cytarabine-21; 6-MP	resistance (3)
**ALL (primary)**							
50	ALL	0.04±0.01 (1)	0.03±0.001 (1)	40.8±4.1 (3)	0.006±0.001 (1)	ALL-2009 (induction 1, 2; consolidation 1); 6-MP	remission (1)
51	ALL	<0.05 (1)	<0.05 (1)	1.5±0.3 (1)	>5 (3)	ALL-2009 (prephase; induction 1, 2); RACOP; COMP; COAP	relapse (2)
52	ALL	>2 (3)	0.7±0.2 (3)	>82 (3)	>5 (3)	ALL-2009 (prephase; induction 1, 2); RACOP; COAP; COMP; HiDAC	resistance (3)
53	ALL	>2 (3)	>2 (3)	>82 (3)	<0.001 (1)	ALL-2009	remission (1)
54	ALL	0.007±0.004 (1)	6.9±0.02 (3)	0.78±0.07 (1)	0.2±0.035 (3)	ALL-2009 (induction 1)	resistance (3)
55	ALL		0.3±0.05 (3)		>5 (3)	ALL-2009 (induction 1)	resistance (3)
56	ALL	>2 (3)	0.4±0.1 (3)	>82 (3)	>5 (3)	ALL-2009 (induction 1, 2; consolidation 1)	resistance (3)
57	ALL	0.8±0.1 (3)	0.5±0.2 (3)	3.2±1.5 (1)	>5 (3)	ALL-2009 (induction 1)	remission (1)
58	ALL	0.2±0.03 (3)	0.05±0.01 (1)	14.3±1 (3)	0.2±0.06 (3)	ALL-2009 (induction 1)	remission (1)
59	ALL		0.1±0.01 (1)	26.3±10.9 (3)	0.007±0.002 (1)	ALL-2009 (induction 1, 2; consolidation 5)	remission (1)
60	ALL	0.01±0.001 (1)	0.05±0.006 (1)	4±1.2 (1)	0.02±0.005 (3)	ALL-2009 (consolidation 5; maintenance)	remission (1)
**ALL (secondary)**							
61	ALL	0.6±0.3 (3)	0.2±0.04 (2)	13.9±3 (3)	>5 (3)	R-CHOEP; R-DHAP; LBM-04 (prephase; block A)	resistance (3)
62	ALL	0.9±0.2 (3)	>2 (3)	>82 (3)	>5 (3)	5+2; 7+3; low-dose cytarabine-14; tyrosine kinase inhibitors	resistance (3)
63	ALL	0.05±0.02 (1)	0.2±0.01 (2)		0.2±0.05 (3)	miniCHOP	resistance (3)
64	ALL		0.9±0.1 (3)	39.4±2.9 (3)		R-FC	remission (1)
65	ALL	0.2±0.04 (3)			7±2.5 (2)	CHOP	resistance (3)
**CLL**							
66	CLL				>0.3 (3)	COP	resistance (3)
67	CLL	0.1±0.02 (1)			0.007±0.003 (1)	miniCHOP; R-miniCHOP	remission (1)
68	CLL	0.8±0.1 (3)			>5 (3)	no	relapse (2)

69	CLL	0.6±0.2 (3)	>5 (3)	R-FC	remission (1)
70	CLL	0.35±0.02 (2)	>5 (3)	chlorambucil	relapse (2)
71	CLL	0.3±0.1 (2)	0.05±0.02 (1)	COP; R-FC	remission (1)
72	CLL	0.2±0.01 (1)	0.02±0.01 (1)	BR	remission (1)
73	CLL	0.3±0.04 (2)	>5 (3)	no	relapse (2)
74	CLL	0.1±0.03 (1)	0.1±0.01 (2)	R-FC	remission (1)
75	CLL	0.5±0.1 (2)	0.04±0.004 (1)	R-FC	remission (1)
76	CLL	0.65±0.2 (3)	0.2±0.08 (3)	chlorambucil	remission (1)
77	CLL	0.4±0.07 (2)	>5 (3)	BR	remission (1)
78	CLL	0.1±0.01 (1)		R-FC	remission (1)
79	CLL	0.3±0.07 (2)	0.1±0.05 (2)	R-FC, BR; CHOP	relapse (2)
80	CLL	1.1±0.3 (3)	0.02±0.005 (1)	R-CHOP; R-CHOEP; R-DHAP; R-FC	resistance (3)
81	CLL	0.1±0.006 (1)	0.04±0.01 (1)	COP; R-FC	remission (1)
82	CLL	0.2±0.1 (1)	>5 (3)	FC; BR; ibrutinib	remission (1)
83	CLL	>1 (3)	>5 (3)	R-CVP; R-FC	remission (1)
84	CLL	0.2±0.02 (1)	>5 (3)	R-COP	resistance (3)
85	CLL	>2 (3)	>0.3 (3)	R-FC; COP	remission (1)
86	CLL	>2 (3)	>5 (3)	R-FC	relapse (2)
87	CLL	0.4±0.1 (2)	0.2±0.07 (3)	R-CHOP; R-CHOEP; R-ESHAP; BR; R-DHAP	relapse (2)
88	CLL	0.2±0.02 (1)		COP; R-FC	remission (1)
89	CLL	0.4±0.07 (2)		R-COP; BR	remission (1)
90	CLL	0.2±0.03 (1)	0.1±0.03 (2)	COP; R-FC	remission (1)
91	CLL	10±1.7 (3)		FC; COP; R-CHOP	resistance (3)
92	CLL	0.1±0.02 (1)	0.05±0.01 (1)	R-FC	remission (1)
93	CLL	0.7±0.1 (3)	0.06±0.03 (2)	R-FC-Lite	remission (1)
94	CLL		>5 (3)	COP; R-FC; COP	resistance (3)
95	CLL		0.3±0.03 (3)	no	relapse (2)
96	CLL	2±0.3 (3)	>5 (3)	R-COP	remission (1)
97	CLL		0.04±0.01 (1)	R-FC	remission (1)
98	CLL	0.2±0.02 (1)	0.2±0.06 (3)	R-FC; R-COP	relapse (2)
99	CLL	0.7±0.04 (3)	0.02±0.005 (1)	COP; FC	resistance (3)

100	CLL	0.1±0.04 (1)			0.06±0.03 (2)	R-COP	remission (1)
101	CLL	0.2±0.06 (1)			0.03±0.006 (1)	R-FC	remission (1)
102	CLL	0.6±0.2 (3)			<0.01 (1)	COP; R-FC	remission (1)
103	CLL				0.01±0.003 (1)	COP; R-FC; R-FC-Lite; R-COP; ibrutinib	remission (1)
104	CLL				>10 (3)	R-COP; R-FC-Lite	remission (1)
105	CLL	0.09±0.01 (1)			0.05±0.008 (1)	COP	remission (1)
106	CLL	0.6±0.3 (3)				COP	remission (1)
107	CLL	0.6±0.1 (3)				COP	resistance (3)
108	CLL	>2 (3)			>5 (3)	R-COP	remission (1)
				**CML**			
109	CML		0.3±0.05 (1)	82±16.5 (2)		tyrosine kinase inhibitors	remission (1)
110	CML			>100 (3)		imatinib	remission (1)
111	CML	0.35±0.08 (2)	0.3±0.1 (1)	38.4±11.7 (1)		no	resistance (3)
112	CML	>2 (3)	1.1±0.3 (3)	124.9±11.2 (3)		low-dose cytarabine-14; dasatinib	remission (1)
113	CML	>2 (3)		>161 (3)		low-dose cytarabine-14	resistance (3)

AIDA: ATRA 45 mg/m2 per os (30 days) + idarubicin 12 mg/m2 intravenously (2, 4, 6, 8 days)

7+3: cytarabine 100-200 mg/m2 intravenously (1-7 days) + daunorubicin 45 mg/m2 intravenously (1-3 days)

7+3+VP: cytarabine 100-200 mg/m2 intravenously (1-7 days) + daunorubicin 45 mg/m2 intravenously (1-3 days) + etoposide 120 mg/m2 intravenously (17-21 days)

5+2: cytarabine 100-200 mg/m2 intravenously once a day (1-5 days) + daunorubicin 45 mg/m2 intravenously (1, 2 days)

6-MP: 6-mercaptopurine 50 mg/m2 intravenously

Low-dose cytarabine-7, -14, -21, -28: 10 mg/m2 subcutaneously twice a day (during 7, 14, 21 or 28 days)

Intermediate-dose cytarabine-7: 600 mg/m2 twice a day (during 7 days)

HAM: high-dose cytarabine 3g/m2 intravenously twice a day (1-3 days) + mitoxantrone 10 mg/m2 intravenously (3-5 days)

HiDAC: high-dose cytarabine 3g/m2 intravenously twice a day (1, 3, 5 days)

High-dose cyclophosphamide: 7 g/m2 intravenously + G-CSF 5 mg/kg intravenously

VAD: vincristine 0.4 mg/m2 intravenously (1-4 days) + adriablastine 10 mg/m2 intravenously (1-4 days) + dexamethasone 40 mg/m2 per os (1-4 days, 11-14 days)

ALL-2009:

- *prephase*: prednisolone 60 mg/m2 per os (1-7 days)
- *induction (phase 1)*: prednisolone or dexamethasone 60 mg/m2 per os or 10 mg/m2 intravenously (8-28 days) + vincristine 2 mg intravenously (8, 15, 22 days) + daunorubicin 45 mg/m2 intravenously (8, 15, 22 days) + L-asparaginase 1000 U/m2 intravenously (29, 36 days) + methotrexate 15 mg, cytarabine 30 mg, dexamethasone 4 mg intrathecally (0, 7, 14, 21, 28, 35 days)

- *induction (phase 2)*: 6-mercaptopurine 25 mg/m2 per os (34-70 days) + cyclophosphamide 1g/m2 intravenously + cytarabine 75 mg/m2 intravenously (45-48, 59-62 days) + L-asparaginase 1000 U/m2 intravenously (50, 57, 64 days) + methotrexate 15 mg, cytarabine 30 mg, dexamethasone 4 mg intrathecally
- *consolidation (phase 1)*: dexamethasone 10 mg/m2 intravenously (71-84 days) + doxorubicin 30 mg/m2 intravenously (72, 85 days) + vincristine 2 mg intravenously (72, 85 days)
- *consolidation (phase 2)*: 6-mercaptopurine 50 mg/m2 per os (92-105 days) + L-asparaginase 10000 U/m2 intravenously (92, 99 days) + methotrexate 15 mg, cytarabine 30 mg, dexamethasone 4 mg intrathecally
- *consolidation (phase 3)*: 6-mercaptopurine 25 mg/m2 per os (106-133 days) + cyclophosphamide 1g/m2 intravenously + cytarabine 75 mg/m2 intravenously (108-111, 122-125 days) + L-asparaginase 10000 U/m2 intravenously (113, 127 days)
- *consolidation (phase 4)*: dexamethasone 30 mg/m2 intravenously (134-136 days) + methotrexate 1.5 g/m2 intravenously + L-asparaginase 10000 U/m2 intravenously
- *consolidation (phase 5)*: dexamethasone 30 mg/m2 intravenously (148-150 days) + cytarabine 2 g/m2 intravenously + L-asparaginase 10000 U/m2 intravenously
- *maintenance*: vincristine 2 mg intravenously (1-3 days) + daunorubicin 45 mg/m2 intravenously (1-3 days) + dexamethasone 10 mg/m2 per os (1-3 days) + 6-mercaptopurine 50 mg/m2 per os (4-28 days) + methotrexate 30 mg/m2 intravenously (2, 9, 16, 23 days) + L-asparaginase 10000 U/m2 intravenously (3, 10 days) + methotrexate 15 mg, cytarabine 30 mg, dexamethasone 4 mg intrathecally (once in three month)

RACOP: daunorubicin 45 mg/m2 intravenously (1-3 days) + cytarabine 100 mg/m2 intravenously twice a day (1-7 days) + cyclophosphamide 400 mg/m2 intravenously (1-7 days) + vincristine 2 mg intravenously (1, 7 days) + prednisolone 60 mg/m2 per os (1-7 days)

COMP: cyclophosphamide 1 g/m2 intravenously (1 day) + vincristine 2 mg intravenously (1 day) + methotrexate 12.5 mg/m2 intravenously (3, 4 days) + prednisolone 100 mg/m2 per os (1-5 days)

COAP: cyclophosphamide 400 mg/m2 intravenously (1 day) + vincristine 2 mg intravenously (1 day) + cytarabine 60 mg/m2 intravenously twice a day (1-5 days) + prednisolone 40 mg/m2 per os (1-5 days)

COP: cyclophosphamide 0.8-1 g/m2 intravenously (1 day) + vincristine 1.4 mg/m2 intravenously (1 day) + prednisolone 60 mg/m2 per os (1-5 days)

R-COP: rituximab 375 mg/m2 intravenously (day 1 during cycle 1) and rituximab 500 mg/m2 intravenously (day 1 during other cycles) + cyclophosphamide 0.8-1 g/m2 intravenously (1 day) + vincristine 1.4 mg/m2 intravenously (1 day) + prednisolone 60 mg/m2 per os (1-5 days)

CHOP: cyclophosphamide 750 mg/m2 intravenously (1 day) + doxorubicin 50 mg/m2 intravenously (1 day) + vincristine 1.4 mg/m2 intravenously (1 day) + prednisolone 40 mg/m2 per os (1-5 days)

R-CHOP: rituximab 375 mg/m2 intravenously (day 1 during cycle 1) and rituximab 500 mg/m2 intravenously (day 1 during other cycles) + cyclophosphamide 750 mg/m2 intravenously (1 day) + doxorubicin 50 mg/m2 intravenously (1 day) + vincristine 1.4 mg/m2 intravenously (1 day) + prednisolone 40 mg/m2 per os (1-5 days)

miniCHOP: cyclophosphamide 600 mg/m2 intravenously (1 day) + doxorubicin 25 mg/m2 intravenously (1 day) + vincristine 1 mg/m2 intravenously (1 day) + prednisolone 40 mg/m2 per os (1-5 days)

R- *miniCHOP*: rituximab 375 mg/m2 intravenously (day 1 during cycle 1) and rituximab 500 mg/m2 intravenously (day 1 during other cycles) + cyclophosphamide 600 mg/m2 intravenously (1 day) + doxorubicin 25 mg/m2 intravenously (1 day) + vincristine 1 mg/m2 intravenously (1 day) + prednisolone 40 mg/m2 per os (1-5 days)

CHOEP: cyclophosphamide 750 mg/m2 intravenously (1 day) + doxorubicin 50 mg/m2 intravenously (1 day) + vincristine 1.4 mg/m2 intravenously (1 day) + prednisolone 40 mg/m2 per os (1-5 days) + etoposide 100 mg/m2 intravenously (1-3 days)

R-CHOEP: rituximab 375 mg/m2 intravenously (day 1 during cycle 1) and rituximab 500 mg/m2 intravenously (day 1 during other cycles) + cyclophosphamide 750 mg/m2 intravenously (1 day) + doxorubicin 50 mg/m2 intravenously (1 day) + vincristine 1.4 mg/m2 intravenously (1 day) + prednisolone 40 mg/m2 per os (1-5 days) + etoposide 100 mg/m2 intravenously (1-3 days)

DHAP: dexamethasone 10 mg/m2 intravenously 4 times a day (1-4 days) + cytarabine 2 g/m2 intravenously twice a day (2 day) + cisplatin 100 mg/m2 intravenously (1 day)

R-DHAP: rituximab 375 mg/m2 intravenously (day 1 during cycle 1) and rituximab 500 mg/m2 intravenously (day 1 during other cycles) + dexamethasone 10 mg/m2 intravenously 4 times a day (1-4 days) + cytarabine 2 g/m2 intravenously twice a day (2 day) + cisplatin 100 mg/m2 intravenously (1 day)

LBM-04:

-prephase: cyclophosphamide 200 mg/m2 intravenously (1-5 day) + dexamethasone 20 mg per os (1-5 day)

- block A: dexamethasone 20 mg per os (1-5 day) + vincristine 2 mg intravenously (1 day) + iphosphamide 800 mg/m2 intravenously (1-5 days) + methotrexate 1.5 g/m2 intravenously (1 day) + cytarabine 150 mg/m2 intravenously (4, 5 days) + vepeside 100 mg/m2 intravenously (4, 5 days) + doxorubicin 25 mg/m2 intravenously (1, 2 days) + methotrexate 15 mg, cytarabine 30 mg, dexamethasone 4 mg intrathecally (1 day)

- block C: dexamethasone 20 mg per os (1-5 day) + vepeside 150 mg/m2 intravenously (3-5 days) + methotrexate 1.5 g/m2 intravenously (1 day) + vinblastine 10 mg intravenously (1 day) + cytarabine 2 g/m2 intravenously (2, 3 days) + methotrexate 15 mg, cytarabine 30 mg, dexamethasone 4 mg intrathecally (1 day)

FC: fludarabine 25 mg/m2 intravenously (1-3 days) + cyclophosphamide 250 mg/m2 intravenously (1-3 days)

R-FC: rituximab 375 mg/m2 intravenously (day 1 during cycle 1) and rituximab 500 mg/m2 intravenously (day 1 during other cycles) + fludarabine 25 mg/m2 intravenously (1-3 days) + cyclophosphamide 250 mg/m2 intravenously (1-3 days)

R-FC-Lite: rituximab 375 mg/m2 intravenously (day 1 during cycle 1) and rituximab 500 mg/m2 intravenously (day 1 during other cycles) + fludarabine 20 mg/m2 intravenously (1-3 days) + cyclophosphamide 150 mg/m2 intravenously (1-3 days)

BR: bendamustine 90 mg/m2 intravenously (1, 2 days) + rituximab 375 mg/m2 intravenously (day 1 during cycle 1) and rituximab 500 mg/m2 intravenously (day 1 during other cycles)

CVP: cyclophosphamide 400 mg/m2 intravenously (1-5 days) + vincristine 1.4 mg/m2 intravenously (1 day) + prednisolone 100 mg/m2 per os (1-5 days)

R-CVP: rituximab 375 mg/m2 intravenously (day 1 during cycle 1) and rituximab 500 mg/m2 intravenously (day 1 during other cycles) + cyclophosphamide 400 mg/m2 intravenously (1-5 days) + vincristine 1.4 mg/m2 intravenously (1 day) + prednisolone 100 mg/m2 per os (1-5 days)

ESHAP: etoposide 60 mg/m2 intravenously (1-4 days) + cisplatin 25 mg/m2 intravenously (1-4 days) + cytarabine 2 g/m2 intravenously (1-4 days) + methylprednisolone 500 mg/m2 intravenously (1-4 days)

R-ESHAP: rituximab 375 mg/m2 intravenously (day 1 during cycle 1) and rituximab 500 mg/m2 intravenously (day 1 during other cycles) + etoposide 60 mg/m2 intravenously (1-4 days) + cisplatin 25 mg/m2 intravenously (1-4 days) + cytarabine 2 g/m2 intravenously (1-4 days) + methylprednisolone 500 mg/m2 intravenously (1-4 days)

Table 4. Immunological markers and cytogenetic abnormalities detected in tumor cells of patients with acute and chronic leukemia with scales for correlation analysis (in parentheses).

Patient#	Diagnosis	Immunological markers	Cytogenetic abnormalities	Therapy response (scale)
		**AML (primary)**		
1	AML	MPO, CD13, CD14, CD15, CD33, CD56, CD64, CD65, HLA-DR	MLL(11q23.3), RELN(q22), TES(q31)	remission (1)
2	AML	MPO, CD13, CD14, CD15, CD33, CD64, CD117, HLA-DR	t(15;17)(q24;q21), P53 (17p13.1)	remission (1)
3	AML	-	Complex changes of karyotype	remission(1)
4	AML	-	Complex changes of karyotype	relapse (2)
5	AML	MPO, CD7, CD13, CD14, CD33, CD34, CD64, CD117, HLA-DR	no	remission(1)
6	AML	MPO, CD13, CD15, CD20, CD33, CD34,CD64, CD65, HLA-DR	no	relapse (2)
7	AML	MPO, CD13, CD33, CD34, CD117, HLA-DR	no	resistance (3)
8	AML	MPO, CD7, CD13, CD19, CDCd34, CD117, HLA-DR	no	remission (1)
9	AML	-	Complex changes of karyotype	resistance (3)
10	AML	MPO, CD7, CD11c, CD13, CD15, CD33, CD64, CD65, CD117, HLA-DR	no	remission (1)
11	AML	MPO, CD5, CD13, CD34, CD117, HLA-DR, TdT	no	resistance (3)
12	AML	MPO, CD13, CD15, CD33, CD64, CD65, CD117, HLA-DR	no	resistance (3)
13	AML	MPO, CD11c, CD13, CD14, CD15, CD20, CD33, CD34, CD64, CD65, HLA-DR	no	remission (1)
14	AML	CD2, CD13, CD20, CD33, CD34, CD64, CD65, CD117	t(15;17)(q24;q21)	remission(1)

15	AML	MPO, CD7, CD33, CD34, CD117, HLA-DR	no	relapse (2)
16	AML	MPO, CD15, CD20, CD33, CD64, CD65, CD117, HLA-DR	MLL(11q23.3)	remission (1)
17	AML	-	no	relapse (2)
18	AML	CD7, CD10, CD13, CD33, CD34, CD71, CD117	no	relapse (2)
19	AML	MPO, CD13, CD14, CD15, CD33, CD64, CD117	t(15;17)(q24;q21)	remission (1)
20	AML	MPO, CD13, CD14, CD15, CD33, CD34, CD64, CD65, CD117	no	relapse (2)
21	AML	-	t(15;17)(q24;q21)	remission (1)
22	AML	-	no	resistance (3)
23	AML	-	no	resistance (3)
24	AML	MPO, CD10, CD13, CD14, CD15, CD33, CD64, CD65	trisomy 8	resistance (3)
25	AML	MPO, CD11c, CD15, CD20, CD25, CD33, CD56, CD64, CD71, CD117, HLA-DR, TdT	trisomy 8, deletion 11	resistance (3)
26	AML	MPO, CD13, CD15, CD33, CD64, CD65, CD117, HLA-DR	no	remission (1)
27	AML	-	no	resistance (3)
28	AML	-	trisomy 9, deletion 11	resistance (3)
29	AML	MPO, CD33, CD34, CD117, HLA-DR	deletion 5, TAS2R1(5p15.31), RELN(q22), TES(q31), P53(17p13.1)	resistance (3)
30	AML	-	no	remission (1)
		**AML (secondary)**		
31	AML	MPO, CD2, CD3, CD4, CD5, CD7, CD8, CD13, CD33, CD34, CD117, HLA-DR	no	resistance (3)
32	AML	CD13, CD34, CD117, GlyA, HLA-DR	i(17)(q11)	resistance (3)
33	AML	-	no	relapse (2)
34	AML	MPO, CD7, CD11c, CD13, CD34, CD117, HLA-DR	deletion 5, RELN(q22), TES(q31)	resistance (3)
35	AML	-	no	relapse (2)

36	AML	-	Complex changes of karyotype	resistance (3)
37	AML	-	trisomy 21	resistance (3)
38	AML	MPO, CD2, CD13, CD15, CD16, CD33, CD34, CD64, CD65, CD117	no	resistance (3)
39	AML	CD13, CD33, CD34, CD56, CD64, CD117, HLA-DR	RELN (q22), TES(q31)	resistance (3)
40	AML	-	no	resistance (3)
41	AML	MPO, CD11c, CD13, CD14, CD15, CD33, CD64, CD65, CD71, HLA-DR	MLL(11q23.3)	relapse (2)
42	AML	-	t(9;22)(q34;q11)	remission (1)
43	AML	CD13, CD33, CD34, CD65, CD117, HLA-DR	no	relapse (2)
44	AML	MPO, CD10, CD33, CD34, CD56, Cd117, HLA-DR	RELN (q22), TES(q31)	resistance (3)
45	AML	-	no	resistance (3)
46	AML	MPO, CD10, CD15, CD33, CD34, CD56, CD117	EVI1(3q26.2), P53(17p13.1)	resistance (3)
47	AML	-	deletion 5, P53(17p13.1)	resistance (3)
48	AML	-	no	relapse (2)
49	AML	MPO, CD7, CD13, CD15, CD33, CD34, CD64, CD65, CD117, HLA-DR	trisomy 8	resistance (3)
		**ALL (primary)**		
50	ALL	CD10, CD15, CD19, CD34, CD65, cCD79a, HLA-DR, TdT	AFF1(4q21), KMT2A(11q23)	remission (1)
51	ALL	CD10, CD19, CD20, CD22, CD34, cCD79a, HLA-DR, TdT	AML1(21q22.12)	relapse (2)
52	ALL	CD10, CD19, CD33, CD34, CD65, cCD79a, HLA-DR, TdT	MLL(11q23.3)	resistance (3)
53	ALL	CD2, cCD3, CD5, CD7	no	remission (1)
54	ALL	CD19, CD34, CD79a, cytIgM, HLA-DR	deletion 9	resistance (3)
55	ALL	cCD3, CD7, CD117	no	resistance (3)
56	ALL	CD10, CD19, CD22, CD33, CD34, cCD79a, HLA-D, TdT	E2A(19p13.3)	resistance (3)

57	ALL	CD10, CD19, CD20, cCD79a, HLA-DR	t(9;22)(q34;q11)	remission (1)
58	ALL	CD10, CD19, CD33, CD34, cCD79a, HLA-DR, TdT	t(9;22)(q34;q11)	remission (1)
59	ALL	CD10, CD19, CD20, CD22, CD34, cCD79a, HLA-DR, TdT	MYB(6q23.3), cMYC(8q24), E2A(19p13.3)	remission (1)
60	ALL	CD2, cCD3, CD4, CD7, CD8, TdT	TRA/D(14q11)	remission (1)
		**ALL (secondary)**		
61	ALL	CD10, CD19, Ki-67	cMYC(8q24), IGH(14q32), t(8;14)(q24;q32)	resistance (3)
62	ALL	CD10, CD19, CD20, CD22, CD34, CD38, CD79a, HLA-DR	trisomy 5, t(9;22)(q34;q11)	resistance (3)
63	ALL	CD20, CD45, Ki-67	no	resistance (3)
64	ALL	CD1a, CD2, CD4, CD7, CD45, Ki-67, TdT	no	remission (1)
65	ALL	CD2, cCD3, CD3, CD5, CD7	no	resistance (3)
		**CLL**		
66	CLL	-	no	resistance (3)
67	CLL	CD5, CD19, CD20, CD23, Ki-67	P53(17p13.1)	remission (1)
68	CLL	CD5, CD19, CD20, CD23, CD43	no	relapse (2)
69	CLL	CD5, CD19, CD20, CD79a, Ki-67	no	remission (1)
70	CLL	CD5, CD19, CD20, CD23, CD43	no	relapse (2)
71	CLL	CD20, CD43, CD45, bcl-2	no	remission (1)
72	CLL	CD20, CD43, CD45, HLA-DR	ATM(11q22.3)	remission (1)
73	CLL	CD20, CD45, bcl-2	no	relapse (2)
74	CLL	-	no	remission (1)
75	CLL	CD3, CD5, CD23, CD45, bcl-2	no	remission (1)
76	CLL	CD5, CD20, CD23, CD45, ZAP-70	no	remission (1)
77	CLL	CD5, CD20, CD23, CD45, bcl-2	no	remission (1)
78	CLL	CD5, CD23, CD45, bcl-2	no	remission (1)
79	CLL	CD5, CD20, CD23, CD43, bcl-2	ATM(11q22.3)	relapse (2)
80	CLL	CD5, CD20, CD23, bcl-2	no	resistance (3)
81	CLL	CD20, CD23, CD45, CD79a, bcl-2, HLA-DR	no	remission (1)

82	CLL	CD5, CD19, CD20, CD23, CD45	P53(17p13.1), trisomy 12	remission (1)
83	CLL	CD5, CD19, CD20, CD23, CD79a, Ki-67	no	remission (1)
84	CLL	CD5, CD19, CD20, CD23	trisomy 12	resistance (3)
85	CLL	CD5, CD19, CD20, CD23, CD45, CD79a, bcl-2, HLA-DR	no	remission (1)
86	CLL	-	trisomy 12	relapse (2)
87	CLL	CD10, CD20, CD23, bcl-2, Ki-67	trisomy 12	relapse (2)
88	CLL	CD19, CD20, CD23, CD45, CD79a, bcl-2, HLA-DR	no	remission (1)
89	CLL	CD5, CD45, bcl-2, HLA-DR	no	remission (1)
90	CLL	CD2, CD3, CD5, CD19, CD20, CD45, bcl-2	no	remission (1)
91	CLL	CD10, CD19, CD20, CD23, CD45, CD79a, bcl-2	no	resistance (3)
92	CLL	CD5, CD20, CD23, CD45, bcl-2	no	remission (1)
93	CLL	CD5, CD20, CD23, CD45, CD79a, bcl-2	no	remission (1)
94	CLL	CD5, CD20, CD23, CD45, ZAP-70	no	resistance (3)
95	CLL	CD5, CD20, CD23, CD43	no	relapse (2)
96	CLL	CD20, CD43, CD79a, bcl-2	no	remission (1)
97	CLL	CD5, CD20, CD23, CD45, bcl-2	ATM(11q22.3)	remission (1)
98	CLL	CD5, CD19, CD20, CD23		relapse (2)
99	CLL	CD23, CD45	no	resistance (3)
100	CLL	CD5, CD10, CD20, CD23, CD43, bcl-2	no	remission (1)
101	CLL	CD20, CD23, CD43, CD79a, bcl-2, HLA-DR	no	remission (1)
102	CLL	CD5, CD19, CD20, CD23, CD38	Complex changes of karyotype	remission (1)
103	CLL	CD5, CD20, CD23, CD45, bcl-2, ZAP-70	ATM(11q22.3), P53(17p13.1)	remission (1)
104	CLL	CD5, CD20, CD23, CD38, CD45, bcl-2, ZAP-70	no	remission (1)
105	CLL	CD10, CD20, CD45, bcl-2, Ki-67	no	remission (1)

106	CLL	CD3, CD20, CD23, CD43, bcl-2, Ki-67	no	remission (1)
107	CLL	CD5, CD20, CD23, CD45, bcl-2, HLA-DR	no	resistance (3)
108	CLL	CD5, CD20, CD23, bcl-2	no	remission (1)
		**CML**		
109	CML	-	t(9;22)(q34;q11)	remission (1)
110	CML	-	t(9;22)(q34;q11)	remission (1)
111	CML	-	t(9;22)(q34;q11)	resistance (3)
112	CML	-	t(9;22)(q34;q11)	remission (1)
113	CML	-	t(9;22)(q34;q11)	resistance (3)

Table 5. Correlation coefficients reflecting the relationships between the drug sensitivity and expression of immunological markers in tumor cells of patients with acute myeloid leukemia.

**CD-markers**	**AML primary**			**AML secondary**		
	**IC_{50} dox.**	**IC_{50} daun.**	**IC_{50} cyt.**	**IC_{50} dox.**	**IC_{50} daun.**	**IC_{50} cyt.**
**Immature markers**						
HLA-DR	-0.25	0.19	0.14	-0.56	0.05	-0.29
TdT	-	0.32	0.15			
CD34	0.01	-0.15	-0.21	0.31	0.24	0.03
**Lymphoid markers**						
**T-cell**						
CD2	0.51	0.16	-0.01	0.42	0.48	0.22
CD3	-	0.38	0.23	-	0.45	0.25
CD5	-	-0.17	-0.44	-	0.34	0.14
CD7	-0.49	0.07	0.05	-0.82	0.38	0.01
CD11c	-0.50	0.41	0.97			
**B-cell**						
CD10	-0.25	-0.20	-0.16	-0.13	-0.29	0.22
CD16				0.50	0.34	-0.25
CD20	0.07	-	0.05			
**Myeloid markers**						
CD13	0.54	-0.34	-0.37	0.27	0.21	-0.37
CD15	-0.41	-0.07	0.10	0.06	0.24	-
CD33	0.25	0.25	0.25	0.13	0.18	-0.07
CD64	-0.12	-0.15	-	0.50	0.32	-0.12
CD65	0.12	-0.11	-	0.54	0.37	-0.21
CD117	0.25	0.32	-0.16	-0.40	-	0.23
**Prognostic markers**						
Ki-67	-	0.35	-	-0.33	0.87	0.33

Red color indicates strong positive correlation ($0.70 \leq r_s \leq 1.00$), blue color indicates moderate positive correlation ($0.30 \leq r_s \leq 0.69$), and green color indicates weak positive correlation ($0.01 \leq r_s \leq 0.29$). Shortening of the dox. denotes doxorubicin, shortening of the daun. denotes daunorubicin, shortening of the cyt. denotes cytarabine.

Table 6. Correlation coefficients reflecting the relationships between the drug sensitivity and cytogenetic abnormalities in tumor cells of leukemia patients.

	**Drug sensitivity of tumor cells of leukemia patients**			
	**IC_{50} dox.**	**IC_{50} daun.**	**IC_{50} cyt.**	**IC_{50} vinc.**
**AML primary**				
P53 (17p13.1)	-	-	0.94	-
**AML secondary**				
P53 (17p13.1)	-0.50	-0.58	0.58	-
RELN (q22)	-	0.33	0.27	-
TES (q31)	-	0.33	0.27	-
EVI (3q26.2)			0.50	-
Translocation t(9;22)(q34;q11)	-	0.71	0.58	-
**ALL primary**				
MLL (11q23.3)	0.29	0.33	0.32	0.22
E2A (19p13.3)	0.45	0.20	0.54	-0.18
cMYC (8q24)	-	-0.45	0.61	-0.45
MYB (6q23.3)	-	-0.58	0.50	-0.58
Translocation t(9;22)(q34;q11)	0.55	0.09	0.09	0.26
**CML**				
Trisomy 6, 7, 8, 17, 21	-	0.58	0.24	-

Red color indicates strong positive correlation (0.70≤r_s≤1.00), blue color indicates moderate positive correlation (0.30≤r_s≤0.69), and green color indicates weak positive correlation (0.01≤r_s≤0.29). Shortening of the dox. denotes doxorubicin, shortening of the daun. denotes daunorubicin, shortening of the cyt. denotes cytarabine, shortening of the vinc. denotes vincristine.

Table 7. Correlation coefficients reflecting the relationships between the drug sensitivity and expression of immunological markers in tumor cells of patients with acute lymphoblastic leukemia.

	**ALL primary**				**ALL secondary**			
	**IC_{50} dox.**	**IC_{50} daun.**	**IC_{50} cyt.**	**IC_{50} vinc.**	**IC_{50} dox.**	**IC_{50} daun.**	**IC_{50} cyt.**	**IC_{50} vinc.**
				**Immature markers**				
HLA-DR	-0.29	-0.40	-0.32	0.50	-	-	-	-
TdT	-0.35	-0.60	-0.29	0.06	-	-	-	-
CD34	-0.16	-0.36	0.08	0.37	0.50	-	1.0	0.50
				**Lymphoid markers**				
				**T-cell**				
CD2	-0.15	-	-0.06	-0.61	0.50	-0.87	0.33	0.50
CD3	-0.15	0.22	-0.06	-0.36	-	-	-	-
CD5	-	0.50	-	-0.50	0.33	-	-	0.33
CD7	-0.15	0.22	-0.06	-0.36	-	-	-	-
				**B-cell**				
CD10	0.55	0.10	0.41	0.45	0.54	0.83	0.56	0.54
CD19	0.06	-0.26	-	0.39	-	1.0	-	-
CD20	-0.06	-0.15	-0.22	0.04	-0.58	0.71	-0.41	-0.58
sCD22	-	0.50	1.0	-0.50	-	-	-	-
CD79a	0.38	-0.10	0.32	0.22	-	-	-	-
				**Myeloid markers**				
CD33	0.59	0.17	0.62	0.53	-	-	-	-

CD117	-	0.33	-	0.27	-	-	-	-
**Prognostic markers**								
Ki-67	-	-	-		-	-1.0	0.50	-
CD45	-0.50	-0.61	-0.33	-0.41	-	0.50	-1.0	-

Red color indicates strong positive correlation (0.70≤r_s≤1.00), blue color indicates moderate positive correlation (0.30≤r_s≤0.69), and green color indicates weak positive correlation (0.01≤r_s≤0.29). Shortening of the dox. denotes doxorubicin, shortening of the daun. denotes daunorubicin, shortening of the cyt. denotes cytarabine, shortening of the vinc. denotes vincristine.

Table 8. Correlation coefficients reflecting the relationships between the drug sensitivity and expression of immunological markers in tumor cells of patients with chronic lymphocytic leukemia.

	**CLL**	
	**IC_{50} doxorubicin**	**IC_{50} vincristine**
**Immature marker**		
HLA-DR	0.43	-0.32
**Lymphoid markers**		
T-cell		
CD5	-	0.10
B-cell		
CD20	0.04	0.23
CD23	0.16	0.07
CD79a	0.24	0.09
**Prognostic markers**		
Ki-67	0.12	0.13
ZAP-70	-	0.61
CD38	0.23	-

Red color indicates strong positive correlation (0.70≤r_s≤1.00), blue color indicates moderate positive correlation (0.30≤r_s≤0.69), and green color indicates weak positive correlation (0.01≤r_s≤0.29).

Table 9. Correlation coefficients reflecting the relationships between the drug sensitivity, *MDR1* mRNA and P-glycoprotein expression in tumor cells of leukemia patients.

	***MDR1* mRNA**	**P-gp**
**AML primary**		
IC_{50} daunorubicin	0.74	-0.31
IC_{50} cytarabine	0.53	-0.52
**AML secondary**		
IC_{50} daunorubicin	0.44	0.62
IC_{50} cytarabine	-	0.25
**ALL primary**		
IC_{50} doxorubicin	-0.19	-0.2
IC_{50} daunorubicin	0.14	-0.51
IC_{50} cytarabine	0.09	0.15
IC_{50} vincristine	0.10	0.06
**CLL**		
IC_{50} doxorubicin	-0.12	-0.53
IC_{50} vincristine	0.25	-0.15

Red color indicates strong positive correlation (0.70≤r_s≤1.00), blue color indicates moderate positive correlation (0.30≤r_s≤0.69), and green color indicates weak positive correlation (0.01≤r_s≤0.29).
